# Uncovering Causal Links Between Dietary Habits and Cardiovascular Diseases

**DOI:** 10.1002/fsn3.70229

**Published:** 2025-05-02

**Authors:** Shaoyu He, Huai Wen, Yicheng Fu, Cheng Chen, Mengchang Xu, Manling Zhang, Mingyi Zhao, Shangping Zhao

**Affiliations:** ^1^ Department of Pediatrics The Third Xiangya Hospital, Central South University Changsha China; ^2^ Hunan Provincial Key Laboratory of the Research and Development of Novel Pharmaceutical Preparations Changsha Medical University Changsha China; ^3^ Department of Critical Medicine Hunan Aerospace Hospital, Hunan Normal University Changsha China

**Keywords:** cardiovascular disease, dietary habits, high‐density lipoprotein cholesterol, mendelian randomization, meta‐analysis, myocardial infarction, stroke

## Abstract

Dietary modification plays a crucial role in preventing cardiovascular diseases (CVDs), but evidence linking specific diets to stroke and acute myocardial infarction (AMI) is limited. This study investigates causal relationships between defined dietary exposures (e.g., fruit/vegetable intake, muesil consumption) and CVD outcomes, while evaluating the potential mediating role of high‐density lipoprotein cholesterol (HDL‐C). We employed two‐sample Mendelian randomization (MR) using genetic data from genome‐wide association studies (GWAS) in the UK Biobank and IEU database, validated with FinnGen data, to examine causal relationships between 83 dietary habits and CVD. Additionally, a meta‐analysis was conducted using studies from PubMed and Web of Science to assess diet‐stroke associations. Random‐effects models were applied to estimate pooled relative risks (RR), with sensitivity analyses for robustness. MR identified eight significant diet‐AMI and eighteen diet‐stroke associations, but HDL‐C did not mediate the diet‐stroke relationship. The meta‐analysis of 50 studies confirmed a link between specific diets and stroke risk. This study confirms associations between specific dietary factors and stroke/AMI, though HDL‐C's role in AMI is unclear. These results reinforce the importance of targeted dietary modifications in primary prevention, and further research is needed to clarify underlying mechanisms.

## Graphical abstract

This study integrates Mendelian randomization and meta‐analysis to investigate causal relationships between specific dietary factors and cardiovascular diseases. Eight dietary traits (e.g., high salt intake and poultry consumption) were linked to elevated acute myocardial infarction (AMI) risk, while full‐cream milk and butter/margarine spreads exhibited protective effects. For stroke, wholemeal bread, soy milk, and moderate cheese/alcohol intake reduced risk, whereas white bread, cornflakes, and “flora + benecol” spreads increased susceptibility. HDL‐C levels may play a mediating role in these associations.

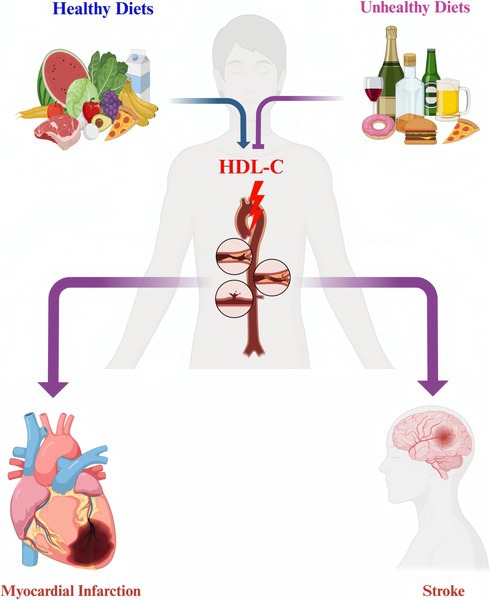

## 1 Introduction

Cardiovascular diseases (CVDs), which encompass a range of disorders affecting the heart and vascular system, remain the leading cause of death worldwide, with rising incidence and poor survival rates despite significant advancements in medical care (Roth et al. 2020). Stroke and myocardial infarction (MI) are the most common CVD events, accounting for nearly 80% of cardiovascular‐related mortality (Sagaro et al. 2021). Both conditions share a common pathophysiology—atherosclerosis—a progressive disease characterized by lipid accumulation and chronic inflammation within arterial walls (Frostegård 2013). In the early stages of atherosclerosis, low‐density lipoprotein (LDL) particles infiltrate damaged endothelial cells, accumulate in the subendothelial space, and trigger an inflammatory response (Feng et al. 2021). This leads to monocyte recruitment and foam cell formation, marking the initial stage of plaque development (Poznyak et al. 2021). Oxidative stress and chronic inflammation further destabilize these plaques, increasing the risk of rupture and thrombosis, which can result in stroke or MI (Frostegård 2013).

Cholesterol metabolism is a critical determinant of CVDs. While LDL contributes to plaque formation, high‐density lipoprotein cholesterol (HDL‐C) offers protective benefits by promoting reverse cholesterol transport, which removes excess cholesterol from arterial walls and transports it to the liver for excretion (Chen et al. 2024; Soppert et al. 2020). HDL mediates cholesterol efflux from peripheral tissues via ABCA1/ABCG1 receptors, a process that reduces atherosclerotic plaque formation and lowers the risk of plaque rupture and thrombosis, key events in CVD progression (Luo et al. 2020). Additionally, HDL inhibits LDL oxidation and suppresses chemokine and adhesion molecule expression through its antioxidant and anti‐inflammatory properties, further impeding atherosclerosis (Akdis 2021). Thus, understanding HDL‐C's role is crucial in CVD prevention, especially when exploring interventions, such as lifestyle modifications, that influence lipid metabolism (Mazidi et al. 2018; Odegaard et al. 2012; Saraf et al. 2022). Emerging evidence suggests that HDL‐C levels can be modified through dietary interventions (Andraski et al. 2019; Morgantini et al. 2018; Zhu et al. 2019). This study also aims to investigate whether HDL‐C mediates the relationship between dietary factors and CVD risk.

Various biological and behavioral factors, including smoking, hypertension, obesity, and dietary habits, contribute to CVD risk (Joseph et al. 2017). These modifiable risk factors can disrupt metabolic processes, accelerating atherosclerosis and increasing CVD risk (Kaminsky et al. 2022). Research shows that modifiable factors account for over 90% of the global CVD burden, with regional differences often linked to dietary patterns (Kaminsky et al. 2022; Organization 2024). Among these, diet has emerged as one of the most cost‐effective and sustainable strategies for CVD prevention (Mendis et al. 2022). In 2017, poor dietary choices were responsible for 58% of CVD‐related disability‐adjusted life years (DALYs), highlighting the critical role of diet in CVD morbidity (Petersen and Kris‐Etherton 2021). Studies have consistently shown that unhealthy diets significantly elevate CVD risk, as demonstrated by higher ischemic stroke rates in Croatia linked to poor diet and other behavioral factors (Odegaard et al. 2012; Pikija et al. 2015). Conversely, adherence to healthy dietary patterns, such as the Mediterranean and plant‐based diets, is associated with a lower incidence of cardiovascular events (Diab et al. 2023; Shay et al. 2013).

While correlations between dietary quality and CVD risk have been well established (Mazidi et al. 2018; Odegaard et al. 2012; Saraf et al. 2022), further research is needed to determine the direct impact of specific dietary components on CVD outcomes. Mendelian randomization (MR) offers a powerful approach for exploring causal relationships between diet and CVD risk by using genetic variants as instrumental variables (Burgess et al. 2015). This method helps mitigate confounding from environmental and behavioral factors, allowing for a clearer assessment of the role of diet in CVD development.

In this study, we applied two‐sample MR analysis using genome‐wide association study (GWAS) data from the UK Biobank, covering 83 dietary habits (Sekula et al. 2016). This was combined with GWAS data on stroke and MI from the IEU database, with further validation in the FinnGen cohort. We also employed a two‐step MR analysis to assess whether HDL‐C mediates the relationship between diet and CVD outcomes. Finally, a meta‐analysis was performed to explore the associations between specific dietary patterns and stroke risk. This research provides robust causal evidence supporting dietary interventions as a key strategy for reducing the global burden of CVD.

## 2 Materials and Methods

### 2.1 Mendelian Randomization Analysis

#### 2.1.1 Study Design

We employed two‐sample Mendelian randomization (MR) and mediation analysis to explore the causal relationship between dietary habits and the risk of stroke and AMI and to assess whether this relationship was mediated by HDL‐C. To ensure the reliability of our findings, we also conducted sensitivity analyses and heterogeneity tests.

In MR studies, three key assumptions are required: association, independence, and exclusion restriction. The instrumental variable must be strongly associated with the exposure (association), independent of confounders that affect both the exposure and outcome (independence), and influence the outcome solely through the exposure without alternative pathways (exclusion restriction). These assumptions ensure valid and reliable causal inference in MR analyses.

#### 2.1.2 Genetic Instrumental Variable (IV) Selection

Single nucleotide polymorphisms (SNPs) significantly associated with the exposure (dietary habits) were identified at the genome‐wide level with a threshold of *p* < 5 × 10^−6^, linkage disequilibrium *r* ^2^ < 0.001, and a distance of 10,000 kb between SNPs to ensure independent signals. To assess the strength of the selected instrumental variables, we calculated the *F*‐statistic using the formula:

$$F=\left[\frac{\left(N-k-1\right)}{k}\right]\times \left[\frac{R^{2}}{1-R^{2}}\right]$$

where *N* represents the sample size, *k* is the number of instrumental variables, and *R* ^*2*^ refers to the proportion of variance in the exposure explained by these instrumental variables. The variance explained by each SNP was calculated as 2 × (1 − MAF) × MAF × β^2^, where MAF is the minor allele frequency and *β* is the effect size of the SNP on the exposure. SNPs with an *F*‐statistic ≥ 10 were considered strong instruments and used in the analysis (Pierce et al. 2011).

#### 2.1.3 Two‐Sample MR Analysis

In this MR study, data on dietary habits was obtained from GWAS conducted by Cole et al. (*n* = 449,210, European ancestry) (Cole et al. 2020). Data for the two outcomes, stroke (cases/controls: 6116/456,817, European descent) and MI (cases/controls: 2321/460,689, European descent), were both obtained from the Family Genome‐Wide Association Studies (GWAS) consortium data summarized in the IEU database, a comprehensive GWAS collecting resource (https://gwas.mrcieu.ac.uk/). Moreover, we also applied GWAS data for stroke (*n* = 439,309) and myocardial infarction (*n* = 406,565) from the FinnGen database to validate the credibility of the results (Kurki et al. 2023) (https://www.finngen.fi/en).

#### 2.1.4 Two‐Step MR Analysis

Genetic data for HDL‐C as a mediator were obtained from the IEU database, which included 403,943 individuals of European descent. First, a two‐sample MR analysis was performed to estimate the causal effect of dietary habits on HDL‐C (denoted as effect (a)). Subsequently, separate two‐sample MR analyses were conducted to estimate the causal effects of HDL‐C on AMI and stroke (denoted as effects (b) and (c), respectively), using the same database. To assess HDL‐C's role as a mediator, the delta method was applied to calculate the indirect effect. The mediating effect ratio was then obtained by dividing the indirect effect by the total effect, providing insights into how much of the relationship between dietary habits and the risk of AMI and stroke is mediated by HDL‐C.

#### 2.1.5 Statistical Analysis

In this study, inverse variance weighted (IVW) regression was employed as the primary method for MR analysis (Rees et al. 2019). A positive effect estimate (*b* > 0) indicates that the exposure (e.g., dietary habits) increases the risk of the outcome (e.g., AMI or stroke), whereas a negative effect estimate (*b* < 0) suggests a protective effect. Statistical significance was determined using a threshold of *p* < 0.05.

Sensitivity analyses were performed to confirm the accuracy of the positive MR findings. Specifically, the MR‐Egger intercept method and MR‐PRESSO test were used to evaluate the presence of horizontal pleiotropy, which occurs when a genetic variant influences multiple traits independently. If horizontal pleiotropy is detected, the MR results may be biased and considered unreliable (Bowden et al. 2015). Heterogeneity across SNP estimates was assessed using Cochran's *Q* test, with a *p* > 0.05 indicating no significant heterogeneity, thus supporting the consistency of the causal estimates (Bowden et al. 2018).

All statistical analyses were conducted using R version 4.4.0, with MR analyses performed using the TwoSampleMR package (https://mrcieu.github.io/TwoSampleMR/). Additionally, meta‐analyses of MR results for the associations between diet and AMI, as well as diet and stroke, were performed using data from the IEU and FinnGen databases, applying a fixed‐effects model. Meta‐analyses were conducted using Stata MP, version 16.0.

### 2.2 Meta‐Analysis

#### 2.2.1 Search Strategy

A systematic literature search was conducted in the PubMed and Web of Science databases to investigate the relationship between diet and stroke, with the final search completed on August 14, 2024. The search strategies for both databases were tailored to their specific formats (Supporting Information File 1: Table S1). This study followed the PRISMA 2020 guidelines for systematic reviews (Supporting Information File 2: Table S1, PRISMA Checklist) (Page et al. 2021), though the protocol was not registered.

#### 2.2.2 Study Selection

After de‐duplication using EndNote 21, two reviewers, Shaoyu He and Yicheng Fu, independently screened the titles and abstracts to exclude irrelevant studies. For ambiguous cases, full‐text reviews were conducted to determine eligibility.

Studies were included if they met the following criteria: (1) prospective design, (2) examined the relationship between dietary consumption and the incidence of stroke, (3) received a Newcastle‐Ottawa Scale (NOS) score of ≥ 7, (4) if multiple studies originated from the same cohort, the one with the larger sample size was retained, and (5) compared different levels of food consumption (e.g., lower versus higher intake) or assessed the extremes of intake (maximum versus minimum) of specific foods.

Exclusion criteria were (1) exposures not directly related to diet (e.g., dietary inflammation index), (2) outcomes unrelated to stroke (e.g., medication intake), (3) studies that did not report relative risk (RR) or hazard ratio (HR) values, and (4) non‐English language publications.

#### 2.2.3 Data Extraction

Study characteristics were compiled into tables by one author (Shaoyu He) and independently verified for accuracy by another author (Yicheng Fu). The results extracted were from the models that adjusted for the greatest number of confounding factors. The extracted data included: author and publication year, country of study, study period, follow‐up duration, final sample size, participants' sex and age, number of stroke cases, type of diet and its subgroups, adjusted relative risk (RR) or hazard ratio (HR) with 95% confidence intervals (CIs), and the confounding factors adjusted for in each study.

#### 2.2.4 Literature Quality Assessment

In this study, the NOS was used to assess the risk of bias in the included prospective cohort studies. The NOS evaluates studies across three domains: selection, comparability, and outcome, with a maximum score of 9 points. Studies scoring 7 or above were considered to be of high quality. Additionally, the certainty of evidence for the outcomes was assessed using the Grading of Recommendations, Assessment, Development, and Evaluation (GRADE) system (Guyatt et al. 2011).

#### 2.2.5 Data Synthesis and Statistical Analysis

A meta‐analysis was conducted when at least two studies provided effect estimates for the same dietary intake. In this analysis, HR were considered equivalent to RR. Initially, results from different subgroups within the same study were pooled using a fixed‐effects model. Subsequently, effect estimates from different studies were synthesized using a random‐effects model. Heterogeneity was assessed using *Q* statistics and the *I* ^2^ index (Higgins and Thompson 2002), with a *p*‐value < 0.05 or an *I* ^2^ value greater than 50% indicating significant heterogeneity. Sensitivity analysis was performed to explore the potential sources of observed heterogeneity. Due to the inclusion of fewer than 10 studies, the Egger test and funnel plot asymmetry were not used to assess publication bias (Sedgwick and Marston 2015). All statistical analyses were performed using Stata MP, version 16.0.

## 3 Results

### 3.1 Two‐Sample MR Analysis

#### 3.1.1 Discovery Results of CVDs in the IEU Database

The MR analysis using the IVW method identified several dietary habits associated with AMI risk, with detailed results available in Supporting Information File 3 (Tables S1 and S2). Certain dietary habits were inversely associated with AMI risk, including: drinking with meals (usually or sometimes) (OR = 0.9954, 95% CI = 0.9924–0.9984, *p* = 0.0026), drinking with meals (usually) (OR = 0.9972, 95% CI = 0.9953–0.9991, *p* = 0.0035), use of butter and margarine spreads (OR = 0.9959, 95% CI = 0.9923–0.9995, *p* = 0.0248), butter spreads alone (OR = 0.9970, 95% CI = 0.9942–0.9998, *p* = 0.0374), whole wheat bread (OR = 0.9975, 95% CI = 0.9952–0.9998, *p* = 0.0340), dried fruit intake (OR = 0.9965, 95% CI = 0.9931–0.9999, *p* = 0.0439), and full‐fat milk (OR = 0.9972, 95% CI = 0.9950–0.9995, *p* = 0.0148). In contrast, other dietary habits were positively associated with increased AMI risk, such as using Flora and Benecol spreads (OR = 1.0197, 95% CI = 1.0056–1.0340, *p* = 0.0062), frequency of salt addition to food (OR = 1.0036, 95% CI = 1.0014–1.0057, *p* = 0.0012), brown bread consumption (OR = 1.0103, 95% CI = 1.0019–1.0189, *p* = 0.0163), total poultry intake (OR = 1.0054, 95% CI = 1.0006–1.0102, *p* = 0.0258), and soy milk consumption (OR = 1.0019, 95% CI = 1.0002–1.0037, *p* = 0.0294).

Eating oily fish (OR = 0.9965, 95% CI = 0.9930–0.9999, *p* = 0.0460), overall alcohol intake (OR = 0.9938, 95% CI = 0.9911–0.9966, *p* = 0.0000), often consuming alcohol during meals (OR = 0.9960, 95% CI = 0.9931–0.9990, *p* = 0.0083), sometimes consuming alcohol during meals (OR = 0.9952, 95% CI = 0.9908–0.9997, *p* = 0.0373), drinking decaffeinated coffee (OR = 0.9948, 95% CI = 0.9906–0.9989, *p* = 0.0128), ingesting soy milk (OR = 0.9681, 95% CI = 0.9443–0.9926, *p* = 0.0110), having whole wheat bread vs. white and brown (OR = 0.9932, 95% CI = 0.9899–0.9964, *p* = 0.0000), whole wheat bread vs. others (OR = 0.9943, 95% CI = 0.9905–0.9980, *p* = 0.0030), red wine (OR = 0.9926, 95% CI = 0.9883–0.9969, *p* = 0.0007), dried fruit consumption (OR = 0.9926, 95% CI = 0.9885–0.9966, *p* = 0.0004), muesli (OR = 0.9902, 95% CI = 0.9854–0.9950, *p* = 0.0001), cereal (OR = 0.9922, 95% CI = 0.9884–0.9961, *p* = 0.0001) and cheese (OR = 0.9921, 95% CI = 0.9885–0.9957, *p* = 0.0000) were all associated with a reduced risk of stroke. In contrast, spreading margarine (OR = 1.0258, 95% CI = 1.0076–1.0443, *p* = 0.0052), spreading flora and benecol (OR = 1.0137, 95% CI = 1.0021–1.0255, *p* = 0.0207), refraining from sugar (OR = 1.0059, 95% CI = 1.0010–1.0108, *p* = 0.0191), refraining from sugar vs. refraining from eggs dairy wheat (OR = 1.0058, 95% CI = 1.0011–1.0106, *p* = 0.0165), eating cornflakes (OR = 1.0085, 95% CI = 1.0023–1.0146, *p* = 0.0067), consuming white bread vs. whole wheat bread (OR = 1.0088, 95% CI = 1.0053–1.0123, *p* = 0.0000), and consuming white bread vs. others (OR = 1.0094, 95% CI = 1.0057–1.0131, *p* = 0.0000) were associated with an increased risk of stroke.

#### 3.1.2 Validation Results of CVDs in the FinnGen Database

To validate the findings, an additional two‐sample MR analysis was performed using GWAS data from the FinnGen database. The detailed results are presented in Supporting Information File 3 (Tables S3 and S4).

The unrestricted intake of sugar, with no limitations on eggs, dairy, or wheat (OR = 1.8419, 95% CI = 1.4435–2.3504, *p* = 0.0000), the use of spreads containing flora and benecol (OR = 21.2770, 95% CI = 5.8849–76.8689, *p* = 0.0000), and the absence of sugar restrictions (OR = 1.7183, 95% CI = 1.3277–2.2238, *p* = 0.0000) are associated with an increased risk of AMI. Other risk factors for AMI included total poultry consumption (OR = 1.5552, 95% CI = 1.1008–2.1971, *p* = 0.0123), white bread instead of wholemeal or brown bread (OR = 1.2003, 95% CI = 1.0185–1.4145, *p* = 0.0294), and skimmed milk compared to other types (OR = 1.4700, 95% CI = 1.0081–2.1437, *p* = 0.0453).

On the other hand, several factors were found to be protective against AMI. These included: the habit of usually drinking with meals plus variability (OR = 0.6008, 95% CI = 0.4769–0.7570, *p* = 0.0000), total cheese consumption (OR = 0.6997, 95% CI = 0.5772–0.8482, *p* = 0.0003), red wine consumption per month (OR = 0.7464, 95% CI = 0.6247–0.8917, *p* = 0.0013), butter spread (OR = 0.5298, 95% CI = 0.3535–0.7941, *p* = 0.0021), cereal type such as muesli (OR = 0.7624, 95% CI = 0.6160–0.9435, *p* = 0.0126), and skimmed, semi‐skimmed, or full cream milk (OR = 0.5969, 95% CI = 0.3893–0.9153, *p* = 0.0180). Similarly, decaffeinated coffee (OR = 0.7983, 95% CI = 0.6622–0.9624, *p* = 0.0182) and the consumption of wholemeal/wholegrain bread (OR = 0.8231, 95% CI = 0.7003–0.9673, *p* = 0.0181), wholemeal/wholegrain bread (OR = 0.8153, 95% CI = 0.6829–0.9733, *p* = 0.0238), and other alcohol intake per month (OR = 0.7000, 95% CI = 0.4963–0.9871, *p* = 0.0419) were associated with a reduced risk of AMI. Additionally, the use of any oil‐based spread compared to never using spreads (OR = 0.7885, 95% CI = 0.6255–0.9940, *p* = 0.0443) and the habit of consuming alcohol with meals (OR = 0.8521, 95% CI = 0.7461–0.9731, *p* = 0.0182) were also identified as protective factors.

White bread instead of other types (OR = 1.3317, 95% CI = 1.1585–1.5309, *p* = 0.0001), or wholemeal/wholegrain/brown bread (OR = 1.2928, 95% CI = 1.1379–1.4689, *p* = 0.0001), as well as other types of milk compared to any other (OR = 7.7839, 95% CI = 1.8202–33.2865, *p* = 0.0056), and the use of spreads containing flora and benecol compared to other spreads (OR = 1.6297, 95% CI = 1.1029–2.4080, *p* = 0.0142) were identified as risk factors for stroke.

On the other hand, protective factors against stroke included the consumption of muesli (OR = 0.6896, 95% CI = 0.5652–0.8415, *p* = 0.0003), total cheese consumption (OR = 0.7912, 95% CI = 0.6877–0.9104, *p* = 0.0011), the habit of usually drinking with meals plus variability among current drinkers (OR = 0.7427, 95% CI = 0.6072–0.9084, *p* = 0.0038), wholemeal/wholegrain bread instead of white or brown bread (OR = 0.8293, 95% CI = 0.7291–0.9431, *p* = 0.0043), any oil‐based spread never used (OR = 0.7956, 95% CI = 0.6548–0.9667, *p* = 0.0214). Additionally, wholemeal/wholegrain bread compared to any other type (OR = 0.8536, 95% CI = 0.7442–0.9791, *p* = 0.0237), other alcohol intake per month (OR = 0.7060, 95% CI = 0.5206–0.9574, *p* = 0.0251), and olive oil spread compared to other spreads (OR = 0.6430, 95% CI = 0.4225–0.9787, *p* = 0.0394) were also associated with a reduced risk of stroke.

#### 3.1.3 Combined Results of CVDs From Meta‐Analysis

**FIGURE 1:**
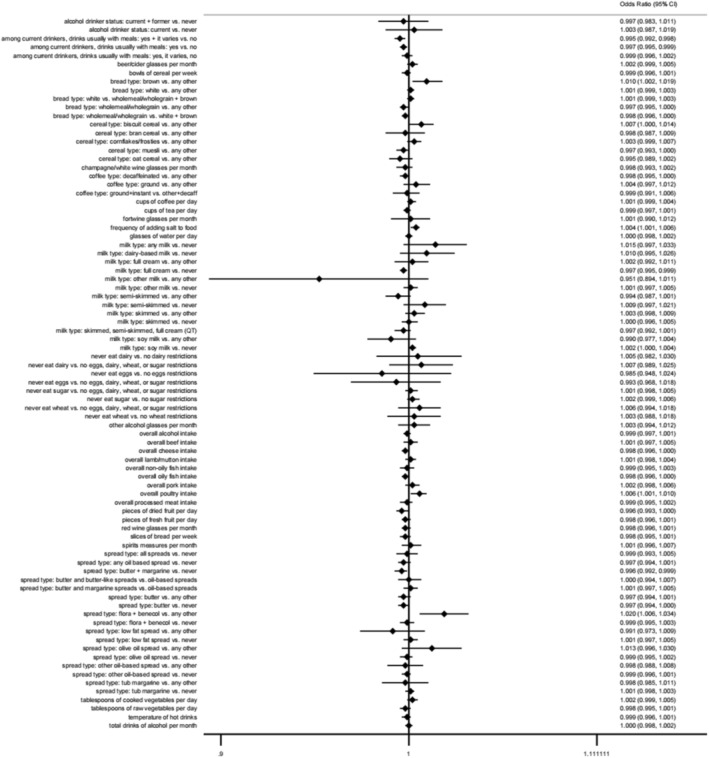
Forest plot of MR analysis: diet and AMI.

As shown in Figure 1, the meta‐analysis results reveal that certain dietary habits are positively associated with an increased risk of AMI. These include the use of “flora + benecol” spreads, consumption of brown bread, the frequency of salt addition to food, and overall poultry intake. Conversely, several dietary habits were found to be protective against AMI. These include the preference for full‐cream milk over no milk consumption, the habit of drinking alcohol specifically with meals among current drinkers, and the use of butter and margarine spreads compared to never using such spreads.

**FIGURE 2:**
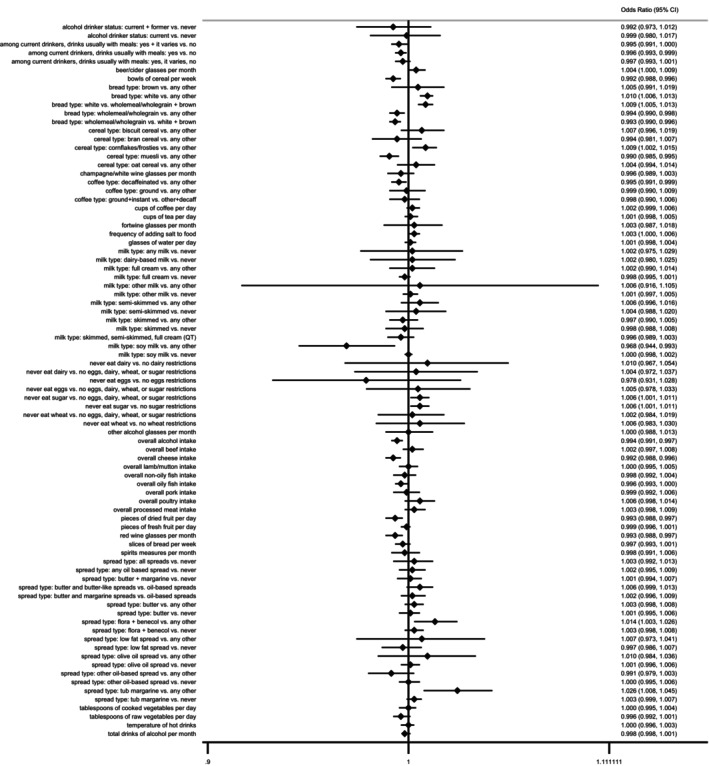
Forest plot of MR analysis: diet and stroke.

As illustrated in Figure 2, several dietary habits are inversely associated with the risk of stroke. These include the consumption of cereal bowls per week, regular alcohol intake with meals, wholemeal/wholegrain bread, muesli cereal, decaffeinated coffee, soy milk, overall alcohol, and cheese intake. Additionally, the daily intake of dried fruit pieces and monthly red wine consumption were also linked to a reduced stroke risk. Conversely, certain dietary preferences were positively associated with an increased risk of stroke. These include preferring white bread over wholemeal/wholegrain or brown bread, choosing cornflakes or frosties cereal over any other type, following strict dietary restrictions such as avoiding sugar, eggs, dairy, or wheat, and opting for spreads like flora and benecol or tub margarine over other types.

### 3.2 Mediation Analysis

Mediation analysis was conducted to determine whether the causal effects of dietary habits on the risk of stroke and AMI were mediated by HDL‐C (Supporting Information File 3: Tables S5–S8). The analysis revealed that, for AMI with HDL‐C as the mediator, the total effects of poultry intake, always drinking with meals, sometimes drinking with meals, butter + margarine versus never, and butter versus never were 0.0054, −0.0026, −0.0046, −0.0041, and −0.0030, respectively. The corresponding mediation effects were 0.0004, −0.0002, −0.0003, −0.0002, and −0.0003, accounting for 7.44%, 7.36%, 6.80%, 5.64%, and 10.41% of the total effects. These findings suggest that HDL‐C may mediate the relationship between poultry meat consumption and alcohol intake with AMI risk. However, no mediation effect of HDL‐C was observed in relation to the risk of stroke for the intake of these foods.

### 3.3 Sensitivity Analysis

Heterogeneity was assessed using the IVW method, while horizontal pleiotropy was evaluated using the MR‐Egger method and MR‐PRESSO test. Evidence of horizontal pleiotropy was identified between dietary habits and HDL‐C, as well as between HDL‐C and both AMI and stroke. This suggests that the instrumental variables may influence the outcomes through pathways other than the intended exposures. The detailed results of the pleiotropy and heterogeneity analyses are presented in Supporting Information File 3 (Tables S9–S12). Overall, the sensitivity analyses indicate that genetic variants may affect the outcomes through multiple biological processes, not solely via the HDL‐C pathway. As such, the findings could be influenced by the pleiotropic effects of the genetic instrumental variables, potentially limiting the representation of true causal effects.

### 3.4 Meta‐Analysis

#### 3.4.1 Literature Search and Study Characteristics

**FIGURE 3:**
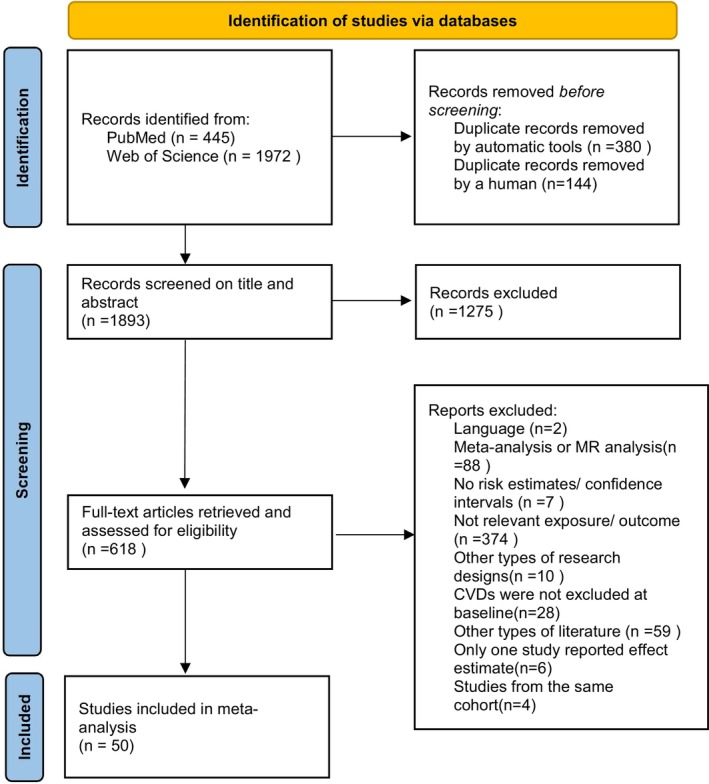
Flowchart of the selection of studies included in the meta‐analysis.

Figure 3 illustrates the flow diagram outlining the comprehensive literature screening process. Based on the eligibility criteria, a total of 60 studies were initially identified as meeting the requirements. However, six studies (Eshak et al. 2012; Kermani‐Alghoraishi et al. 2024; Li et al. 2024; Liu et al. 2000; Yang et al. 2022; Yu et al. 2016) were excluded because they analyzed relevant dietary habits within the scope of a single publication, and four others (Colditz 1990; Liu et al. 2019; Oude Griep et al. 2011; Pacheco et al. 2024) were excluded for examining the same dietary habits from the same cohort. Ultimately, 50 studies were included in the meta‐analysis, covering a variety of food categories: carbohydrates (Eshak et al. 2014; Juan et al. 2017; Muraki et al. 2015), beverages (Bernstein et al. 2012; Grobbee et al. 1990; Janzi et al. 2020; Kokubo et al. 2013; Lopez‐Garcia et al. 2009; Makita et al. 2012; Pacheco et al. 2020; Stampfer et al. 1988; Tanabe et al. 2008), fruits and vegetables (Gao et al. 2021; Johnsen et al. 2003; Larsson et al. 2013; Larsson and Wolk 2016; Lee et al. 2019; Miller et al. 2017; Pacheco et al. 2022; Scheffers et al. 2019; Yoshizaki et al. 2020; Yu et al. 2023), nuts (Guasch‐Ferre et al. 2017; Ivey et al. 2021; Larsson et al. 2018), meat and eggs (Abdollahi et al. 2019; Amiano et al. 2016; Bonaccio et al. 2017; Cui et al. 2022; Djousse and Gaziano 2008; Grau et al. 2022; He et al. 2002; Hu et al. 1999; Iso et al. 2001; Larsson et al. 2015; Larsson et al. 2011a, 2011b; Mohammadifard et al. 2022; Morris et al. 1995; Wallin et al. 2018; Ward et al. 2020), dairy (Buendia et al. 2018; Dalmeijer et al. 2013; Larsson et al. 2012; Praagman et al. 2015; Talaei et al. 2019; Tanno et al. 2021), and other categories (Buijsse et al. 2010; Dong et al. 2017; Tong et al. 2020).

The included studies had follow‐up periods ranging from 3.3 to 30 years and were published between 1988 and 2023. Geographically, the studies originated from various regions, including the United States (*n* = 18), China (*n* = 2), Japan (*n* = 8), Iran (*n* = 3), Europe (*n* = 18), and multiple countries and regions (*n* = 1). A detailed overview of the study characteristics is provided in Supporting Information File 1: Table S2.

#### 3.4.2 Bias Risk and Evidence Quality

Supporting Information File 1: Table S3 provides the quality assessment results. Of the 50 studies included, 18 received a score of 7, 27 received a score of 8, and 5 received a score of 9, indicating generally high quality. The overall quality of evidence for each outcome is presented in Supporting Information File 1: Table S4. The primary reason for downgrading the evidence level was indirectness, as most of the studies reviewed were conducted in Western countries, potentially limiting the generalizability of the findings to other populations.

#### 3.4.3 Results

As shown in Table 1, the meta‐analysis results indicate that the intake of fruits (RR = 0.92, 95% CI: 0.89–0.96), cereal (RR = 0.92, 95% CI: 0.87–0.98), coffee (RR = 0.81, 95% CI: 0.74–0.90), cheese (RR = 0.92, 95% CI: 0.87–0.97), fermented dairy (RR = 0.94, 95% CI: 0.90–0.98), and milk (RR = 0.95, 95% CI: 0.92–0.99) were associated with a reduced incidence of stroke. In contrast, the consumption of sugar‐sweetened beverages (SSBs) (RR = 1.19, 95% CI: 1.09–1.30) was associated with an increased incidence of stroke.

**TABLE 1:** Meta‐analysis results.

Food group	Food item	Number of studies	Risk estimate for cardiovascular disease unless otherwise specified	Reference
Carbohydrate	Rice	2	1.02 (0.93–1.12) *p* = 0.663 (*I* ^2^ = 0.0%, *p* = 0.767)^a^	Eshak et al. (2014), Muraki et al. (2015)
	Cereal	2	0.92 (0.87–0.98) *p* = 0.007 (*I* ^2^ = 42.1%, *p* = 0.189)^a^	Juan et al. (2017), Tong et al. (2020)
Meat & eggs	Fish	8	0.99 (0.94–1.04) *p* = 0.736 (*I* ^2^ = 3.1%, *p* = 0.406)^a^	Bonaccio et al. (2017), Cui et al. (2022), He et al. (2002), Iso et al. (2001), Morris et al. (1995), Tong et al. (2020), Wallin et al. (2018), Ward et al. (2020)
	Red meat	5	1.00 (0.86–1.16) *p* = 0.966 (*I* ^2^ = 72.5%, *p* = 0.006)^a^	Amiano et al. (2016), Grau et al. (2022), Larsson et al. (2011a, 2011b), Tong et al. (2020)
	Eggs	6	1.07 (1.00–1.14) *p* = 0.043 (*I* ^2^ = 0.0%, *p* = 0.552)^a^	Abdollahi et al. (2019), Djousse and Gaziano (2008), Hu et al. (1999), Larsson et al. (2015), Mohammadifard et al. (2022), Tong et al. (2020)
Fruits & vegetables	Fruits	8	0.92 (0.89–0.96) *p* = 0.000 (*I* ^2^ = 11.5%, *p* = 0.340)^a^	Gao et al. (2021), Johnsen et al. (2003), Larsson et al. (2013), Miller et al. (2017), Pacheco et al. (2022), Scheffers et al. (2019), Tong et al. (2020), Yoshizaki et al. (2020)
	Vegetables	7	0.97 (0.93–1.04) *p* = 0.636 (*I* ^2^ = 49.8%, *p* = 0.063)^a^	Johnsen et al. (2003), Larsson et al. (2013), Larsson and Wolk (2016), Lee et al. (2019), Miller et al. (2017), Tong et al. (2020), Yoshizaki et al. (2020)
	Legume	3	0.84 (0.65–1.09) *p* = 0.202 (*I* ^2^ = 71.3%, *p* = 0.031)^a^	Miller et al. (2017), Tong et al. (2020), Yu et al. (2023)
Beverages	Alcohol	2	0.93 (0.46–1.90) *p* = 0.848 (*I* ^2^ = 68.0%, *p* = 0.077) ^1^	Makita et al. (2012), Stampfer et al. (1988)
	Coffee	3	0.81 (0.74–0.90) *p* = 0.000 (*I* ^2^ = 0.0%, *p* = 0.535)^a^	Grobbee et al. (1990), Kokubo et al. (2013), Lopez‐Garcia et al. (2009)
	Green tea	2	0.62 (0.30–1.28) *p* = 0.198 (*I* ^2^ = 86.0%, *p* = 0.008)^a^	Kokubo et al. (2013), Tanabe et al. (2008)
	SSBs^b^	3	1.19 (1.09–1.30) *p* = 0.000 (*I* ^2^ = 0.0%, *p* = 0.925)^a^	Bernstein et al. (2012), Janzi et al. (2020), Pacheco et al. (2020)
Dairy	Cheese	4	0.92 (0.87–0.97) *p* = 0.001 (*I* ^2^ = 0.0%, *p* = 0.651) ^a^	Dalmeijer et al. (2013), Larsson et al. (2012), Praagman et al. (2015), Tong et al. (2020)
	Other fermented dairy	5	0.94 (0.90–0.98) *p* = 0.002 (*I* ^2^ = 0.0%, *p* = 0.515)^a^	Buendia et al. (2018), Dalmeijer et al. (2013), Larsson et al. (2012), Praagman et al. (2015), Tong et al. (2020)
	Milk	5	0.95 (0.92–0.99) *p* = 0.008 (*I* ^2^ = 0.0%, *p* = 0.799)^a^	Larsson et al. (2012), Praagman et al. (2015), Talaei et al. (2019), Tanno et al. (2021), Tong et al. (2020)
Nuts & other	Nuts	4	0.93 (0.84–1.03) *p* = 0.150 (*I* ^2^ = 52.6%, *p* = 0.096)^a^	Guasch‐Ferre et al. (2017), Ivey et al. (2021), Larsson et al. (2018), Tong et al. (2020)
	Chocolate	2	0.73 (0.44–1.21) *p* = 0.223 (*I* ^2^ = 72.1%, *p* = 0.058)^a^	Buijsse et al. (2010), Dong et al. (2017)

^a^ The results of the Cochran's *Q* test.

^b^ Sugar‐sweetened beverages.

In the meta‐analysis, heterogeneity was reduced after excluding certain individual studies. Specifically, removing the study by Yu et al. (2023) from the legume category and Ivey et al. (2021) from the nut category eliminated the heterogeneity in these groups. Additionally, excluding the study by Grau et al. (2022) from the red meat category reduced heterogeneity to 17.1%. These exclusions indicate that the removed studies had a significant influence on the heterogeneity of the meta‐analysis results. The sensitivity analysis findings are presented in Supporting Information File 1: Figure S1, and the forest plot results from the meta‐analysis are shown in Supporting Information File 1: Figure S2.

## 4 Discussion

This study provides crucial insights into how specific dietary habits affect the risk of CVDs, particularly AMI and stroke. By integrating MR and meta‐analysis, our research identified several key findings: (1) the consumption of “flora + benecol” spreads, brown bread, frequent salt addition, and poultry intake was associated with an increased risk of AMI; (2) full‐cream milk, alcohol consumption with meals, and the use of butter and margarine spreads were linked to a reduced risk of AMI; **(3)** for stroke, protective dietary factors included wholemeal/wholegrain bread, muesli, decaffeinated coffee, soy milk, and moderate alcohol and cheese intake, while white bread, cornflakes, and flora and benecol spreads increased stroke risk. These results underscore the pivotal role of dietary choices in influencing cardiovascular risk and offer significant insights for refining dietary guidelines to better prevent CVDs.

One of the most important contributions of this study is its challenge to the traditional view of HDL‐C as a key mediator in diet‐related CVD risk. Our findings showed no significant mediation effect of HDL‐C on the relationship between dietary habits and the risk of AMI and stroke. This challenges the conventional view that increasing HDL‐C levels offers broad protection against CVDs (Barter and Rye 1996; Kaneko et al. 2021). While many studies have linked higher HDL‐C levels to reduced risks of stroke and heart attack (Meilhac 2015; Morvaridzadeh et al. 2024; Qie et al. 2021; Sergi et al. 2024), our results suggest that this relationship is more complex. The presence of pleiotropy—where genetic variants influence both HDL‐C and other pathways—may contribute to this complexity. Thus, the traditionally accepted protective role of HDL‐C in cardiovascular health may warrant re‐evaluation.

Although this study found no significant mediating effect of HDL‐C levels on diet‐related cardiovascular risk, it does not diminish the critical role of HDL particles in cardiovascular health. Previous research suggests that HDL's compositional structure, beyond its levels, also influences cardiovascular outcomes (von Eckardstein et al. 2023). HDL particle size and protein composition vary, and under pathological conditions such as chronic kidney disease or coronary artery disease, changes in HDL apolipoprotein composition can impair its antioxidant function, even converting HDL into a pro‐inflammatory phenotype (Marsche et al. 2013). Therefore, the lack of a mediating effect of HDL‐C levels in this study may reflect these complexities. This aligns with evidence from randomized controlled trials, which show that simply elevating HDL‐C levels pharmacologically does not reduce cardiovascular events (Schwartz et al. 2012). These findings suggest that while HDL‐C levels may serve as a useful biomarker in normolipidemic populations, a more comprehensive approach, including subclassification of HDL based on its composition (e.g., apolipoprotein content, lipidome profiles), is needed for broader clinical applications. Overall, HDL‐C concentration alone is an insufficient predictor of cardiovascular risk, and strategies targeting HDL function, particularly enhancing cholesterol efflux and anti‐inflammatory properties, remain promising for CVD prevention. Future studies could use multi‐omics MR to further explore the impact of diet on the HDL function–CVD pathway.

The study also provides further clarity on the complex relationship between alcohol consumption and cardiovascular health. Our findings align with previous research suggesting that moderate alcohol intake, particularly when consumed with meals, is associated with a reduced risk of AMI and stroke (Ronksley et al. 2011). The mechanisms by which alcohol exerts these protective effects likely involve multiple pathways, including the enhancement of HDL‐C levels, fibrinolysis, and prevention of LDL oxidation (Hillbom and Numminen 1998; Sato et al. 2002; Suter and Vetter 1999). However, it is essential to recognize that alcohol's protective effect is non‐linear; excessive consumption is known to elevate CVD risk and lead to other chronic health issues (Biddinger et al. 2022). Consequently, promoting moderate alcohol consumption as a preventive strategy should be approached with caution due to the potential for harm when consumed in excess.

The study also sheds light on the cardiovascular effects of dairy products. Cheese and milk, particularly rich in calcium, were associated with a reduced risk of stroke, likely due to calcium's protective effects on vascular health (Adebamowo et al. 2015). Moreover, fermented dairy products like cheese may contain bioactive compounds that provide protection against atherosclerotic cardiovascular disease (ASCVD) (Şanlier et al. 2017). Similarly, wholemeal bread, cereals, and muesli, high in dietary fiber, were linked to a reduced risk of CVDs (Threapleton et al. 2013). Fiber is known to improve lipid profiles and reduce CVD risk, reinforcing the benefits of these foods. Muesli's inclusion of dried fruits, which have demonstrated cardiovascular benefits (Xu et al. 2019), further supports its protective role in cardiovascular health. Furthermore, a systematic review indicated that nut intake has no effect on HDL‐C (Del Gobbo et al. 2015), which is consistent with our study results.

Interestingly, our MR analysis found that poultry consumption was associated with an increased risk of AMI. This finding contrasts with some existing literature and highlights the need for further investigation (Del Gobbo et al. 2015; Iqbal et al. 2021; van den Brandt 2019). Variations in meat processing methods and consumption patterns may account for these differences, suggesting that the relationship between poultry intake and CVD risk is complex and warrants more detailed exploration (Del Gobbo et al. 2015; Iqbal et al. 2021; van den Brandt 2019).

Our findings regarding sugar‐sweetened beverages (SSBs) also support existing evidence that SSB consumption increases stroke risk (Malik and Hu 2019). SSBs contribute to cardiometabolic risk factors such as insulin resistance, obesity, and elevated triglyceride levels, all of which are significant contributors to CVDs (Saraf et al. 2022). Limiting SSB consumption should remain a priority in public health interventions, particularly as global obesity rates continue to rise (Saraf et al. 2022).

The role of dairy products in cardiovascular health remains debated. While some studies suggest benefits, particularly from fermented dairy (Fontecha et al. 2019; Lovegrove and Hobbs 2016), others associate dairy intake with increased cardiovascular mortality (Mazidi et al. 2019). These discrepancies may stem from dairy's complex nutritional composition, which includes both beneficial components like unsaturated fatty acids, amino acids, and probiotics, and potentially harmful ones like saturated fatty acids (Ma et al. 2024). Probiotics in dairy products can positively influence lipid metabolism and gut microbiota, while the mineral profile, especially calcium‐potassium‐magnesium, may help regulate blood pressure by modulating vascular tone and sodium balance (Chrysant and Chrysant 2013). Milk fat, with its diverse fatty acids, can promote cardiovascular health by reducing inflammation and regulating metabolism (Torres‐Gonzalez and Rice Bradley 2023). However, some saturated fats, such as myristic acid, may increase LDL cholesterol, complicating the effects of dairy on cardiovascular health (Givens 2023; McCarty and DiNicolantonio 2016). Given the varied mechanisms at play, future research should categorize dairy products based on their nutritional profiles and examine both their beneficial and harmful effects.

This study has several limitations. First, the GWAS data used for MR primarily come from European populations, limiting the generalizability of our findings to other ethnic groups. Dietary patterns vary across regions, with differences in macronutrient and micronutrient intake that correspond to disparities in CVD incidence. Thus, further validation in diverse populations is needed. Second, while MR‐Egger intercept and MR‐PRESSO tests were used to assess pleiotropy, some evidence of pleiotropy was detected, which may influence the results. Horizontal pleiotropy suggests that SNPs could affect the outcome through other pathways, potentially distorting the causal association or generating false‐positive relationships. Although MR‐PRESSO was used to identify and remove outliers, horizontal pleiotropy persisted. Future studies should consider selecting more suitable instrumental variables to address this challenge. Third, the limited number of studies included in the meta‐analysis for certain food items may have reduced statistical power and introduced heterogeneity. Finally, this study focused only on prospective studies, which may not account for all confounding factors. Future research should perform separate meta‐analyses for individual food items to draw more specific and reliable conclusions.

## 5 Conclusions

This study offers valuable insights into how specific food choices influence CVD risk. By integrating MR and meta‐analysis, we provide strong evidence supporting causal relationships between dietary choices and cardiovascular outcomes. The findings highlight the importance of food‐based strategies for CVD prevention and management, underscoring the need for evidence‐based dietary guidelines to reduce the global burden of CVDs. As CVDs remain the leading cause of death worldwide, this research emphasizes the urgency of developing targeted, actionable nutritional interventions tailored to diverse populations and individual risk profiles. The insights from this study can inform public health initiatives, leading to more effective dietary interventions and shaping future health policies. Ultimately, this research contributes to alleviating the societal and economic burden of cardiovascular diseases and promoting healthier global communities.

## Author Contributions

**Shaoyu He:** conceptualization (equal), data curation (equal), formal analysis (equal), investigation (lead), resources (equal), software (lead), visualization (equal), writing – original draft (lead). **Huai Wen:** investigation (equal), validation (equal), visualization (equal), writing – original draft (lead). **Yicheng Fu:** conceptualization (lead), data curation (equal), methodology (equal), writing – review and editing (equal). **Cheng Chen:** writing – review and editing (equal). **Mengchang Xu:** writing – review and editing (equal). **Manling Zhang:** writing – review and editing (equal). **Mingyi Zhao:** conceptualization (equal), funding acquisition (lead), project administration (equal), supervision (equal). **Shangping Zhao:** supervision (equal).

## Ethics Statement

This study is based on publicly available summarized data. Ethical approval and informed consent had been obtained in all original studies.

## Consent

Informed consent had been obtained from all individual participants in all original studies.

## Conflicts of Interest

The authors declare no conflicts of interest.

## Supporting information


Table S1.

Data S1.

Data S2.

## Data Availability

The summary data of IEU can be downloaded from the website “https://gwas.mrcieu.ac.uk/”. The summary data of FinnGen can be downloaded from the website “https://www.finngen.fi/en/access_results”. The summary data of diet habits can be downloaded from the website “http://biobank.ndph.ox.ac.uk/showcase/”.
