# Supplementary material for: Uncovering Causal Links Between Dietary Habits and Cardiovascular Diseases
Source: Food Sci Nutr. 2025 May 2;13(5):e70229. doi: 10.1002/fsn3.70229 (PMC12048706; doi:10.1002/fsn3.70229)
Supplement: Supplementary file 1 — Table S1. [file FSN3-13-e70229-s003.docx]

**Table S1** Search strategy for Web of Science and PubMed

Web of Science

| # | Searches |
| --- | --- |
| 1 | TS= ("Stroke" OR "intracranial hemorrhages" OR "cerebral hemorrhage" OR "ischemic stroke" OR "hemorrhagic stroke" OR "subarachnoid hemorrhage" OR "Cerebrovascular Accident" OR "Cerebral Stroke" OR "Brain Vascular Accident" OR "Cerebrovascular Stroke") |
| 2 | TS= ("Eating" OR "food intake" OR "Diet" OR "diets" OR "dietary") |
| 3 | TS= ("Prospective Studies" OR "prospective cohort study") |
| 4 | 1 AND 2 AND 3 |

PubMed

| # | Searches |
| --- | --- |
| 1 | "Stroke"[Mesh] |
| 2 | ("Stroke"[Mesh]) OR ("intracranial hemorrhages"[Title/Abstract] OR "cerebral hemorrhage"[Title/Abstract] OR "ischemic stroke"[Title/Abstract] OR "hemorrhagic stroke"[Title/Abstract] OR "subarachnoid hemorrhage"[Title/Abstract] OR "Cerebrovascular Accident"[Title/Abstract] OR "Cerebral Stroke"[Title/Abstract] OR "Brain Vascular Accident"[Title/Abstract] OR "Cerebrovascular Stroke"[Title/Abstract]) |
| 3 | "Eating"[Mesh] |
| 4 | ("Eating"[Mesh]) OR ("food intake"[Title/Abstract] OR "Diet"[Mesh] OR diets[Title/Abstract] OR dietary[Title/Abstract]) |
| 5 | "Prospective Studies"[Mesh] |
| 6 | ("Prospective Studies"[Mesh]) OR ("prospective cohort study"[Title/Abstract]) |
| 7 | 2 AND 4 AND 6 |

| **Table S2** Characteristics of Studies Included in the Meta-Analysis | | | | | | | |
| --- | --- | --- | --- | --- | --- | --- | --- |
| Author (Year) | country | Study period, Follow up (year) | Participants n, sex, Age (Years), number of cases/deaths | Exposure vs. comparator, subgroup | Outcome | RR or HR（95% CIs） | Adjustment |
| Juan Juan et al. (2017) | America | cohort 1: 1984, 26 years follow-up cohort 2: 1986, 24 years follow-up | 114573 M/F, age 30-75 years: 2458 ischemic stroke cases | categorical intake variables, Whole grain cold breakfast cereal: < 1 serving/month vs. > 1 serving/week | IS inc. | 0.88 (0.80, 0.96) | age, BMI, smoking status, alcohol intake, physical activity, family history of diabetes, cancer and heart disease, multivitamin use, hypertension, high cholesterol at baseline, total energy intake and modified alternative health eating index score, menopausal status and postmenopausal hormone use |
| I. Muraki et al. (2015) | America | cohort 1: 1984, 26 years follow-up cohort 2: 1991, 20 years follow-up cohort 3: 1986, 24 years follow-up | 207556 M/F, age 42-59 years: 4672 stroke cases | Rice intake, servings/wk: <1 vs. ≥5, Total rice | stroke inc. | 1.04 (0.89, 1.21) | age, sex, cohort, ethnicity, BMI, smoking status, alcohol intake, physical activity, family history of myocardial infarction, menopausal status and postmenopausal hormone use, oral contraceptive use, multivitamin use, current aspirin use, prevalent hypertension, prevalent hypercholesterolemia, prevalent diabetes, total energy intake and modified alternate Healthy Eating Index score |

| Author (Year) | country | Study period, Follow up (year) | Participants n, sex, Age (Years), number of cases/deaths | Exposure vs. comparator, subgroup | Outcome | RR or HR（95% CIs） | Adjustment |
| --- | --- | --- | --- | --- | --- | --- | --- |
| E. S. Eshak et al. (2014) | Japan | cohort 1: 1990-2009 cohort 2: 1993-2007, 15-18 years follow-up | 91223 M/F, age 40-69 years: 4395 stroke cases | daily rice consumption: Low vs. High | stroke inc. | 1.01 (0.90-1.14) | age; sex; public health center area; history of hypertension; history of diabetes; use of lipid-lowering drugs; BMI; smoking status; ethanol intake; leisure-time sports activity; occupation; intakes of seafood, meat, fruit, vegetables, soy, SFAs, and sodium; total energy intake and menopausal status and hormone use |
| A. M. Bernstein et al. (2012) | America | cohort 1: 1980, 28 years follow-up  cohort2:1986, 22 years follow-up | cohort 1: 84085 F, age 30-55 years: 2938 stroke cases, cohort 2: 43371 M, age 40-75 years: 1416 stroke cases | Soda consumption: None vs. Once per day or more, Sugar-sweetened soda | stroke inc. | 1.16 (1.00-1.34) | age, calendar time and included red meat, poultry, fish, whole-fat dairy products, low-fat dairy products, and nuts and simultaneously controlled for intakes of cereal fiber, alcohol, fruit and vegetable intake, and trans unsaturated fatty acids and other potential nondietary confounding variables. These variables were updated biennially and included physical exercise, cigarette smoking, menopausal status in women, parental history of early myocardial infarction, years of multivitamin use, vitamin E supplement use, aspirin use at least once per week, total energy and BMI |

| Author (Year) | country | Study period, Follow up (year) | Participants n, sex, Age (Years), number of cases/deaths | Exposure vs. comparator, subgroup | Outcome | RR or HR（95% CIs） | Adjustment |
| --- | --- | --- | --- | --- | --- | --- | --- |
| L. S. Pacheco et al. (2020) | America | 1995-1996, 20 years follow-up | 106178 F, age 52.1± 13.4 years: 5258 stroke cases | SSB Consumption: Rare/Never vs. ≥1 Serving Per d | stroke inc. | 1.21 (1.04–1.41) | age, race/ethnicity, socioeconomic status, smoking status, alcohol intake, cardiovascular disease family history, physical activity, aspirin use, menopausal status, menopausal hormone therapy use, history of hypertension, BMI, and total energy intake |
| S. Janzi et al. (2020) | Sweden | 1991-1996, 19.5 years follow-up (mean) | 25877 M/F, age 45-74 years old: 2580 stroke cases | Sugar-Sweetened Beverages: <1 serving/week vs. >8 servings/week | stroke inc. | 1.19 (1.01-1.40) | age, sex, season of dietary assessment, diet method, energy intake, smoking status, educational level, leisure-time physical activity, alcohol consumption, BMI, and dietary habits including intake of processed meat, coffee, saturated fatty acids, and fiber density |

| Author (Year) | country | Study period, Follow up (year) | Participants n, sex, Age (Years), number of cases/deaths | Exposure vs. comparator, subgroup | Outcome | RR or HR（95% CIs） | Adjustment |
| --- | --- | --- | --- | --- | --- | --- | --- |
| N. Tanabe et al. (2008) | Japan | 1998: 5 years follow-up | 6358 M/F, age 40-89 years: 110 stroke cases | Green tea consumption: Low vs. High | stroke inc. | 0.41 (0.24–0.70) | age, sex, history of hypertension, diabetes, presence of medically treated disease, BMI, systolic blood pressure, serum total cholesterol, smoking habit, cigarettes/day, daily intake of grain products, vegetable intake, salted vegetable intake, fruit intake, balance of meat and fish intake, daily intake of soybean paste soup, weekly intake of milk, amount of time spent walking each day, consumption of another type of tea, green or roasted, consumption of black or oolong tea and consumption of coffee |
| Y. Kokubo et al. (2013) | Japan | 1995, 1998, 13 years follow-up (mean) | 81978 M/F, age 45-74 years: 3425 stroke cases | Green Tea Consumption: None vs. 2–3 Cups/d | stroke inc. | 0.86 (0.78–0.95) | age, sex, smoking, alcohol, BMI, history of diabetes mellitus, medication of antihypercholesterolemia and antihypertension, sports, dietary intake of fruits, vegetables, fish, and energy, public health centers and coffee or green tea consumption |
|  |  |  |  | Coffee Consumption: None vs. ≥2 Cups/d | stroke inc. | 0.81 (0.72–0.91) |  |

| Author (Year) | country | Study period, Follow up (year) | Participants n, sex, Age (Years), number of cases/deaths | Exposure vs. comparator, subgroup | Outcome | RR or HR（95% CIs） | Adjustment |
| --- | --- | --- | --- | --- | --- | --- | --- |
| E. Lopez-Garcia et al. (2009) | America | 1980, 24 years follow-up | 83076 F, age 30-55 years: 2280 stroke cases (426 HS cases, 1224 IS cases) | Caffeinated coffee consumption, cups: <1/mo vs. ≥4/d, total stroke | stroke inc. | 0.85 (0.69-1.06) | age, smoking status, BMI, physical activity, alcohol intake, menopausal status and use of hormone replacement therapy, aspirin use, total caloric intake, quintiles of calcium, potassium, sodium, and folate intake, glycemic load, whole grain intake, tertiles of fruits, vegetables, fish consumption, high blood pressure, hypercholesterolemia, and type 2 diabetes mellitus |
| D. E. Grobbee et al. (1990) | America | 1986, 2 years follow-up | 45589 M, age 40-75 years: 54 stroke cases | Coffee Consumption: None vs. ≥4 Cups/d | stroke inc. | 0.48 (0.18-1.31) | age, quintiles of Quetelet's index, smoking habits, history of diabetes mellitus, quintiles of alcohol use, parental history of myocardial infarction, specific health profession, quintiles of dietary intake of energy, cholesterol, and saturated, monounsaturated and polyunsaturated fat |
| M. J. Stampfer et al. (1988) | America | 1980, 4 years follow-up | 87526 F, age 34-59 years: 120 stroke cases (66 IS cases) | Alcohol intake: 0g/day vs. ≥15.0g/day | IS inc. | 0.60 (0.30-1.30) | history of infarction before the age of 60, menopausal status and hormone use, period, smoking status, hypertension, diabetes, high cholesterol, age, obesity, exercise, cholesterol intake, saturated - fat intake, and polyunsaturated - fat intake |

| Author (Year) | country | Study period, Follow up (year) | Participants n, sex, Age (Years), number of cases/deaths | Exposure vs. comparator, subgroup | Outcome | RR or HR（95% CIs） | Adjustment |
| --- | --- | --- | --- | --- | --- | --- | --- |
| S. Makita et al. (2012) | Japan | 2002-2005, 5.5 years follow-up (mean) | 8014 M, age 40-80 years: 186 IS cases | Daily alcohol consumption: 0.1g/day vs. 49.7g/day | IS inc. | 1.26 (0.87-1.83) | age, sex, smoking status and pack-years of smoking, education, BMI, total physical activity, aspirin use, history of hypertension, diabetes, family history of myocardial infarction, and intakes of total energy, alcohol, coffee, fresh red meat, processed meat and fish |
| Q. Gao et al. (2021) | Japan | cohort 1: 1995-2009 cohort 2: 1998-2012, 13.1 years follow-up (median) | 87177 M/F, age 40-69 years: 4091 stroke cases (2557 cerebral infarction cases, 1516 haemorrhagic stroke cases and 18 unknown subtype cases) | Intakes of total fruits: Low vs. High, M | stroke inc. | 0.90 (0.79-1.03) | sex, study area, BMI, medication use for hyper tension and hypercholesterolemia, diabetes history, occupation, drinking status, smoking status and sports level |
|  |  |  |  | Intakes of total fruits: Low vs. High, F | stroke inc. | 0.85 (0.73-0.99) |  |

| Author (Year) | country | Study period, Follow up (year) | Participants n, sex, Age (Years), number of cases/deaths | Exposure vs. comparator, subgroup | Outcome | RR or HR（95% CIs） | Adjustment |
| --- | --- | --- | --- | --- | --- | --- | --- |
| F. R. Scheffers et al. (2019) | Netherlands | 1993-1997, 14.6 years follow-up (mean) | 34560 M/F, age 20-69 years: 145 stroke cases | Quintiles of fruit consumption: Low vs. High | stroke inc. | 0.93 (0.73-1.19) | age, sex, educational level, physical activity, smoking, alcohol consumption, DHD15-index and fruit consumption or pure fruit juice consumption, possible intermediate factors and important CVD risk factors |
| L. S. Pacheco et al. (2022) | America | cohort 1: 1986, 30 years follow-up | cohort 1: 68786 F, age 40-75 years cohort 2: 41701M, age 30-55 years: 5290 stroke cases | Avocado Intake: Never or <1 per month vs. ≥2 per week | stroke inc. | 0.94 (0.78–1.14) | age, race, ancestry, alcohol intake, smoking status, physical activity, family history of diabetes, family history of myocardial infarction, family history of cancer, baseline diabetes, baseline hypertension or antihypertensive medication use, baseline hypercholesterolemia or cholesterol‐lowering medication use, multivitamin use, aspirin use, postmenopausal status and menopausal hormone use, total energy intake, BMI, red and processed meat, fruits and vegetables, nuts, soda, whole grains, eggs, tortilla, breads, cheese intakes and trans‐fat |

| Author (Year) | country | Study period, Follow up (year) | Participants n, sex, Age (Years), number of cases/deaths | Exposure vs. comparator, subgroup | Outcome | RR or HR（95% CIs） | Adjustment |
| --- | --- | --- | --- | --- | --- | --- | --- |
| T. Yoshizaki et al. (2020) | Japan | 1995-1998, 14 years follow-up | 16498 M/F, age 40-69 years: 839 stroke cases | Total vegetable consumption: Low vs. High | stroke inc. | 1.18 (0.98-1.43) | age, sex, and study area, alcohol intake, cigarette smoking status, type of work, self-reported perceived mental stress, BMI, metabolic equivalent task-hours per day, quartiles of energy intake, energy-adjusted dietary consumption of fish, meat, and sodium, past history of diabetes, treatment of hypertension, and treatment of hypercholesterolemia |
|  |  |  |  | Total fruit consumption: Low vs. High | stroke inc. | 0.89 (0.74-1.06) |  |
| S. C. Larsson et al. (2013) | Sweden | 1998, 10.2 years follow-up (mean) | 74961 M/F, age 50-71 years: 4089 stroke cases | Total fruit consumption: Low vs. High | stroke inc. | 0.87 (0.78–0.97) | age, sex, smoking status and pack-years of smoking, education, body mass index, total physical activity, aspirin use, history of hypertension, diabetes, family history of myocardial infarction, and intakes of total energy, alcohol, coffee, fresh red meat, processed meat and fish |
|  |  |  |  | Total vegetable consumption: Low vs. High | stroke inc. | 0.90 (0.80–1.01) |  |
| S. P. Johnsen et al. (2003) | Denmark | 1993-1997, 3.09 years follow-up (median) | 54506 M/F, age 50-64 years: 266 stroke cases | Fruit intake: Low vs. High | IS inc. | 0.60 (0.38- 0.95) | sex, total energy intake, smoking status, systolic blood pressure, diastolic blood pressure, total serum cholesterol, history of diabetes, BMI, alcohol intake, intake of red meat, intake of n-3 polyunsaturated fatty acids, physical activity and education |
|  |  |  |  | Vegetable intake: Low vs. High | IS inc. | 1.00 (0.66- 1.53) |  |

| Author (Year) | country | Study period, Follow up (year) | Participants n, sex, Age (Years), number of cases/deaths | Exposure vs. comparator, subgroup | Outcome | RR or HR（95% CIs） | Adjustment |
| --- | --- | --- | --- | --- | --- | --- | --- |
| V. Miller et al. (2017) | North America and Europe, South America, the Middle East, south Asia, China, southeast Asia, and Africa | 2003-2013, 7.4 years follow-up (median) | 135335 M/F, age 35-70 years: 2234 stroke cases | Fruit intake: <3 per week vs. >3 per day | stroke inc. | 0.95 (0.78-1.15) | age, sex, centre, energy intake, current smoker, diabetes, urban/rural location, physical activity, education level, and tertiles of white meat, red meat, and intake of breads and cereals |
|  |  |  |  | Vegetable intake: 1 per day vs. >3 per day | stroke inc. | 1.09 (0.89-1.34) |  |
|  |  |  |  | Legume intake: <1 per month vs. >1 per day | stroke inc. | 0.88 (0.69-1.12) |  |
| S. C. Larsson et al. (2016) | Sweden | 1997,13 years follow-up | 69313 M/F, age 45-83 years: 4531 stroke cases | Total potato consumption: 0–3.4 times/wk vs. >7 times/wk, total stroke | stroke inc. | 0.94 (0.84, 1.04) | age, education, family history of MI before 60 y of age, smoking status and pack-years of smoking, aspirin use, walking or bicycling, exercise, BMI, history of hypertension, history of hypercholesterolemia, alcohol consumption, total energy intake, DASH diet score and sex |

| Author (Year) | country | Study period, Follow up (year) | Participants n, sex, Age (Years), number of cases/deaths | Exposure vs. comparator, subgroup | Outcome | RR or HR（95% CIs） | Adjustment |
| --- | --- | --- | --- | --- | --- | --- | --- |
| D. H. Lee et al. (2019) | America | 1986, 26 years follow-up | 110680 M/F, age < 53 years: 4278 stroke cases | Frequency of mushroom consumption (per serving): Never or almost never vs. Per 2/wk increase | stroke inc. | 1.02 (0.99, 1.05) | age, total calorie intake, smoking status, BMI, physical activity, race, family history of myocardial infarction, baseline high blood cholesterol, baseline high blood pressure, alcohol intake, multivitamin use, Prudent dietary pattern, Western dietary pattern, menopausal status and postmenopausal hormone use |
| S. Yu et al. (2023) | China | 2012-2013, 4.66 years follow-up (median): no report cases | 2477 M/F, age >60 years: NA | soybean consumption: Rare vs. ≥4 times/week | stroke inc. | 0.48 (0.28-0.84) | age, gender, race, educational status, sleep duration, annual income, current smoking, current drinking, physical activity, baseline systolic blood pressure, diastolic blood pressure, BMI, low density lipoprotein cholesterol, fasting plasma glucose, eGFR, total cholesterol, triglyceride, uric acid |
| S. C. Larsson et al. (2018) | Sweden | 1997, 17 years follow-up | 61364 M/F, age 45-83 years: 3782 stroke cases | Frequency of nut consumption: None vs. ≥3/week | IS inc. | 1.03 (0.80-1.32) | age, sex, education, family history of myocardial infarction before 60 years of age, smoking, walking/bicycling, exercise, aspirin use and consumption of alcohol, fruits, vegetables, total energy, BMI, history of diabetes, history of hypertension and history of hypercholesterolaemia |

| Author (Year) | country | Study period, Follow up (year) | Participants n, sex, Age (Years), number of cases/deaths | Exposure vs. comparator, subgroup | Outcome | RR or HR（95% CIs） | Adjustment |
| --- | --- | --- | --- | --- | --- | --- | --- |
| K. L. Ivey et al. (2021) | America | 2011-2018, 3.5 years follow-up (median) | 179827 M/F, age >20 years: 3641 stroke cases | Frequency of nut intake: <1/Month vs. ≥5/Week | stroke inc. | 0.81 (0.72-0.92) | age, age*age, sex, race, BMI, smoking status, frequency of alcohol intake, level of physical activity, level of education and the modified DASH score |
| M. Guasch-Ferre et al. (2017) | America | cohort 1:1980-2012, 28.7 years follow-up (mean) cohort 2 :1991-2013, 21.5 years follow-up cohort 3:1986-2012, 22.5 years follow-up | 210836 M/F, age 25-75 years: 5910 stroke cases | Frequency of nut consumption: Never or almost never vs. Five or more Times per week | stroke inc. | 0.98 (0.86–1.13) | age, caucasian, BMI, physical activity, smoking status, physical examination for screening purposes, current multivitamin use, current aspirin use, family history of diabetes mellitus, myocardial infarction or cancer, history of hypertension or hypercholesterolemia, intake of total energy, alcohol, red or processed meat, fruits, and vegetables, menopausal status and hormone use |
| H. Iso et al. (2001) | America | 1980, 14 years follow-up | 79839 F, age 30-55 years: 574 stroke cases | Average Frequency of Fish Intake, Total stroke:＜1 per Month vs. ≥5 per Week | stroke inc. | 0.48 (0.21-1.06) | age, smoking, time interval, Joules, BMI, alcohol intake, menopausal status and postmenopausal hormone use, vigorous exercise, usual asprin use, multivitamin use, history of hypertension, frequency of total fruit and vegetable servings and for nutrient intake of saturated fat, trans-unsaturated fat, linoleic acid, animal protein and calcium |

| Author (Year) | country | Study period, Follow up (year) | Participants n, sex, Age (Years), number of cases/deaths | Exposure vs. comparator, subgroup | Outcome | RR or HR（95% CIs） | Adjustment |
| --- | --- | --- | --- | --- | --- | --- | --- |
| K. He, et al. (2002) | America | 1986, 12 years follow-up | 43671 M, age 40-75 years: 608 stroke cases | Fish intake: <1/mo vs. ≥5/wk | stroke inc. | 0.83 (0.52-1.29) | BMI, physical activity, history of hypertension, smoking status, use of aspirin, fish oil and multivitamins, intake of total calories, total fat, saturated fat, trans-unsaturated fat, alcohol, potassium, and magnesium, total servings of fruits and vegetables and hypercholesterolemia at baseline |
| M. C. Morris et al. (1995) | America | 1983, 4 years follow-up | 21185 M, age 40-84 years: 173 stroke cases | Weekly fish consumption: <1 vs. ≥5 | stroke inc. | 0.7 (0.3-1.5) | each level of fish consumption, age, aspirin and beta-carotene assignment, smoking, alcohol consumption, obesity, diabetes mellitus, vigorous exercise, parental history of myocardial infarction before age 60 years, history of hypertension, history of hypercholesterolemia, vitamin supplement use and saturated fat intake |
| M. Bonaccio et al. (2017) | Italy | 2005-2010, 4.3 years follow-up (median) | 20969 M/F, age ≥35 years: 66 stroke cases | Fish intake (times/week): 0-1.99 vs. ≥4 | stroke inc. | 0.62 (0.26–1.51) | age, sex, energy intake, education, smoking, drugs for diabetes, drugs for hypertension, drugs for lipids, MDS without fish, blood glucose, LDL-cholesterol and low-grade inflammation |

| Author (Year) | country | Study period, Follow up (year) | Participants n, sex, Age (Years), number of cases/deaths | Exposure vs. comparator, subgroup | Outcome | RR or HR（95% CIs） | Adjustment |
| --- | --- | --- | --- | --- | --- | --- | --- |
| S. Cui et al. (2022) | China | 2016-2019, 4.56 years follow-up (median) | 57701 M/F, age 20-74 years: 807 stroke cases | Fish Consumption (g/week): Less than 300 vs. More than 600 | stroke inc. | 1.00 (0.79–1.26) | age, sex, educational levels, marital status, retirement status, smoking status, alcohol drinking status, physical activity levels, sleep qualities, obesity status, dietary energy intakes, consumption of fruit, vegetables, peanuts, wholegrains, processed and unprocessed meats, bean products, salt and oil, smoking status, alcohol drinking status, physical activity levels, sleep qualities, obesity status, dietary energy intakes and consumption of fruit, vegetables, peanuts, wholegrains, processed and unprocessed meats, bean products, salt and oil |
| R. E. Ward et al. (2020) | America | 2011-2017, 3.3 years follow-up (median) | 197761 M/F, age 66± 12 years (mean): 4042 IS cases | Fish intake: <1/month vs. ≥5 servings/week | IS inc. | 1.13 (0.93–1.38) | age, sex race, BMI, education, smoking status, alcohol intake and exercise |

| Author (Year) | country | Study period, Follow up (year) | Participants n, sex, Age (Years), number of cases/deaths | Exposure vs. comparator, subgroup | Outcome | RR or HR（95% CIs） | Adjustment |
| --- | --- | --- | --- | --- | --- | --- | --- |
| A. Wallin et al. (2018) | Sweden | 1998, 11.8 years follow-up (mean) | 2225 M/F, age 45-84 years: 321 stroke cases | Fish intake: ＞3/week vs. 3/month | stroke inc. | 1.04(0.66-1.64) | attained age, sex, time since diabetes diagnosis, BMI, physical activity, education, cigarette smoking, total energy intake, alcohol, history of high cholesterol, history of hypertension and DASH diet component score |
| P. Amiano et al. (2016) | Spain | 1992-1996, 13.8 years follow-up (mean) | 41020 M/F, age 29-69 years: 674 stroke cases (531 IS cases, 79 HS cases) | Unprocessed red meat consumption (g/day): Low vs. High, M | stroke inc. | 0.81 (0.54-1.21) | age, centre, total energy, BMI, waist circumference, smoking status, smoking before 20 years of age, recreational physical activity, educational level, alcohol consumption, use of vitamin supplements, use of antithrombotic or antihaemorrhagic agents, use of cardiovascular drugs, use of salicylic acid or derivatives, incident acute myocardial infarction cases, diabetes, self-reported diseases, menopausal status, hormone replacement therapy and oral contraceptives, energy from carbohydrates, protein and fats, and intakes of vegetables, fruit and dairy products and fish |
|  |  |  |  | Unprocessed red meat consumption (g/day): Low vs. High, F | stroke inc. | 1.21 (0.79-1.85) |  |
|  |  |  |  | Processed red meat consumption (g/day): Low vs. High, M | stroke inc. | 0.92 (0.64- 1.32) |  |
|  |  |  |  | Processed red meat consumption (g/day): Low vs. High, F | stroke inc. | 0.81 (0.51- 1.27) |  |

| Author (Year) | country | Study period, Follow up (year) | Participants n, sex, Age (Years), number of cases/deaths | Exposure vs. comparator, subgroup | Outcome | RR or HR（95% CIs） | Adjustment |
| --- | --- | --- | --- | --- | --- | --- | --- |
| S. C. Larsson et al. (2011), M | Sweden | 1997, 10.1 years follow-up (mean) | 40291 M, age 45-79 years: 2409 stroke cases | Red meat consumption (g/d): <62.5 vs. ≥136.2 | stroke inc. | 1.15 (1.00, 1.33) | age, smoking status, pack-years of smoking, education, BMI, total physical activity, histories of diabetes and hypertension, aspirin use, family history of myocardial infarction and intakes of total energy, alcohol, fish, fruit and vegetables |
| S. C. Larsson et al. (2011), F | Sweden | 1997, 10.4 years follow-up (mean) | 34670 F, age 49-83 years: 1680 stroke cases | Red Meat Consumption (g/d): <36.5 vs. ≥86 | stroke inc. | 1.12 (0.95–1.32) | age, smoking status and pack-years of smoking, education, BMI, total physical activity, history of diabetes, history of hypertension, aspirin use, family history of myocardial infarction and intake of total energy, alcohol, coffee, fish, fruits, and vegetables |
| N. Grau et al. (2022) | Iran | 2001, 11.2 years follow-up (median) | 5432 M/F, age ≥35 years: 157 stroke cases | Red meat intake: Low vs. High | stroke inc. | 0.49 (0.31–0.77) | age, sex, education, smoking status, daily physical activity, family history of cardiovascular disease, diabetes mellitus, hypertension, hypercholesterolaemia, aspirin use, body mass index, dietary factor including processed meat, fish, fruit and vegetable, hydrogenated and non-hydrogenated vegetable oil, fast food, cereals, legumes, animal fats, sweets, soft drink and beverages and nut |

| Author (Year) | country | Study period, Follow up (year) | Participants n, sex, Age (Years), number of cases/deaths | Exposure vs. comparator, subgroup | Outcome | RR or HR（95% CIs） | Adjustment |
| --- | --- | --- | --- | --- | --- | --- | --- |
| F. B. Hu et al. (1999) | America | cohort 1: 1986-1994, 8 years follow-up cohort2:1980-1994, 14 years follow-up | cohort 1: 37851 M, age 40-75 years: 258 stroke cases, cohort 2: 80082 F, age 30-55 years: 563 stroke cases | Amount of Eggs Consumed:<1 Per week vs. ≥1 Per day, M | stroke inc. | 1.07 (0.66-1.75) | total energy intake, smoking, alcohol consumption, history of hypertension, parental history of MI, BMI; and current multivitamin use, and vitamin E supplement use, physical activity in metabolic equivalents per week, regular vigorous exercise and menopausal status and postmenopausal hormone use |
|  |  |  |  | Amount of Eggs Consumed:<1 Per week vs. ≥1 Per day, F | stroke inc. | 0.89 (0.60-1.31) |  |
| N. Mohammadifard et al. (2022) | Iran | 2001, 11.3 years follow-up (median) | 4367 M/F, age 50.8 ± 11.7 years: 125 stroke cases | Egg intake: <1 time/wk vs. ≥2 times/wk | stroke inc. | 0.99 (0.59-1.69) | age, sex, education, residency, smoking status, daily physical activity, family history of cardiovascular disease, aspirin, BMI and Global Dietary Index |

| Author (Year) | country | Study period, Follow up (year) | Participants n, sex, Age (Years), number of cases/deaths | Exposure vs. comparator, subgroup | Outcome | RR or HR（95% CIs） | Adjustment |
| --- | --- | --- | --- | --- | --- | --- | --- |
| S. C. Larsson et al. (2015) | Sweden | 1997, 13 years follow-up | 70571 M/F, age 45-79 years: 4299 stroke cases | Egg consumption: 0-3/mo vs. ≥1/d | IS inc. | 0.95 (0.77-1.17) | sex, age and includes education, family history of myocardial infarction before 60 y of age, smoking status and pack-years of smoking, aspirin use, walking/bicycling, exercise, BMI, history of hypertension, hypercholesterolemia, and diabetes, and intakes of total energy, alcohol, fruit and vegetables, and processed meat |
|  |  |  |  |  | HS inc. | 1.03 (0.65-1.64) |  |
| L. Djousse et al. (2008) | America | 1981, 20 years follow-up (mean) | 21327 M, age 40-85 years: 1342 stroke cases | Egg consumption: <1/week vs. 7+/week | stroke inc. | 0.99 (0.80 – 1.23) | age, BMI, smoking, history of hypertension, vitamin intake, alcohol consumption, vegetable consumption, breakfast cereal, physical activity, treatment arm atrial fibrillation, diabetes mellitus, hypercholesterolemia, and parental history of premature myocardial infarction |
| A. M. Abdollahi et al. (2019) | Finland | 1950-1984, 21.2± 7.2 years follow-up | 1950 M, age 42-60 years: 217 stroke cases | Egg consumption: <15 g/d vs. >45 g/d | stroke inc. | 0.81 (0.54-1.23) | age, examination year, energy intake, BMI, pack-years of smoking, leisure-time physical activity, hypertension medication and intakes of alcohol and fruit, berries and vegetables |

| Author (Year) | country | Study period, Follow up (year) | Participants n, sex, Age (Years), number of cases/deaths | Exposure vs. comparator, subgroup | Outcome | RR or HR（95% CIs） | Adjustment |
| --- | --- | --- | --- | --- | --- | --- | --- |
| G. W. Dalmeijer et al. (2013) | Netherlands | 1993-1997, 13 years follow-up | 33625 M/F: 531 stroke cases | Fermented dairy | stroke inc. | 0.92 (0.83–1.01) | gender, age, total energy intake, physical activity, smoking, education, BMI, intake of ethanol, coffee, fruit, vegetables, fish, meat and bread |
|  |  |  |  | Cheese | stroke inc. | 0.96 (0.88–1.06) |  |
| J. Praagman et al. (2015) | Netherlands | 1990-1993, 17.3 years follow-up (median) | 4235 M/F, age ≥55 years: 564 stroke cases | Total milk: <200 vs. >400 | stroke inc. | 0.91 (0.72–1.15) | age, gender, total energy intake, BMI, smoking, education level, alcohol intake, intakes of vegetables, fruit, meat, bread, fish, coffee and tea |
|  |  |  |  | Fermented dairy: <50 vs. >100 | stroke inc. | 1.08 (0.87–1.34) |  |
|  |  |  |  | Cheese: <20 vs. >40 | stroke inc. | 0.96 (0.75–1.22) |  |
| K. Tanno et al. (2021) | Japan | 2002- 2005, 10.7 years follow-up (mean) | 14121 M/F, age 40-69 years: 478 stroke cases | Milk Intake Frequency: <2 Cups/Week vs. ≥12 Cups/Week, M | stroke inc. | 0.97 (0.59–1.61) | age, smoking status, alcohol consumption status, exercise habits, fruits and vegetables intake frequency and the ratio of total fish and soy products consumption to meat consumption, BMI, systolic blood pressure, glycated haemoglobin, total cholesterol, high-density lipoprotein cholesterol, use of antihypertensives and menopausal state |

| Author (Year) | country | Study period, Follow up (year) | Participants n, sex, Age (Years), number of cases/deaths | Exposure vs. comparator, subgroup | Outcome | RR or HR（95% CIs） | Adjustment |
| --- | --- | --- | --- | --- | --- | --- | --- |
|  |  |  |  | Milk Intake Frequency: <2 Cups/Week vs. ≥12 Cups/Week, F | stroke inc. | 1.03 (0.68–1.55) |  |
| M. Talaei et al. (2019) | Iran | 2001, 10.9 years follow-up (median) | 5432 M/F, age >35 years: 141 stroke cases | Frequency of whole milk intake: None vs. Daily and more | stroke inc. | 0.95 (0.38–2.38) | age, sex, educational level, BMI, physical activity, smoking status, dietary intakes of red meat, poultry, fish, vegetables, fruit, legumes, tea, coffee, and non-diet cola and baseline diabetes and hypertension |
| J. R. Buendia et al. (2018) | America | cohort 1:1980, 30 years follow-up cohort 2: 1986, 24 years follow-up | cohort 1: 55898 M/F, age 30-55 years:1749 stroke cases cohort 2: 17984 M/F, age 40-75 years:517 stroke cases | Yogurt intake: <1/month vs. ≥2/week | stroke inc. | 0.91 (0.79–1.04) | age, race, smoking, family history of MI, physical activity, BMI, BP-lowering medication use and intakes of total energy, alcohol, trans fatty acids, fiber, milk and cheese |

| Author (Year) | country | Study period, Follow up (year) | Participants n, sex, Age (Years), number of cases/deaths | Exposure vs. comparator, subgroup | Outcome | RR or HR（95% CIs） | Adjustment |
| --- | --- | --- | --- | --- | --- | --- | --- |
| S. C. Larsson et al. (2012) | Sweden | 1997, 10.2 years follow-up (mean) | 74961 M/F, age 50-71 years: 4089 stroke cases | Milk: Low vs. High | stroke inc. | 0.90 (0.82–1.00) | age, sex, smoking status, education, BMI, total physical activity, aspirin use, history of hypertension, diabetes, family history of myocardial infarction, and intakes of total energy, alcohol, coffee, fresh red meat, processed meat, fish, fruits and vegetables |
|  |  |  |  | Sour milk and yogurt: Low vs. High | stroke inc. | 0.98 (0.90–1.08) |  |
|  |  |  |  | Cheese | stroke inc. | 0.91 (0.81–1.01) |  |
| T. Y. N. Tong et al. (2020) | Europe | 1992-2000, 12.7 years follow-up (mean) | 418329 M/F, age 40-70 years: 7378 stroke cases (4281 IS cases, 1430 HS cases) | Red meat | IS inc. | 1.07 (0.96-1.20) | age, smoking status and number of cigarettes per day, history of diabetes, prior hypertension, prior hyperlipidaemia, Cambridge physical activity index, employment status, level of education completed, current alcohol consumption, BMI and calibrated intake of energy |
|  |  |  |  | White fish | IS inc. | 0.98 (0.94-1.03) |  |
|  |  |  |  | Fatty fish | IS inc. | 1.00 (0.95-1.06) |  |
|  |  |  |  | Milk | IS inc. | 0.93 (0.89-0.97) |  |
|  |  |  |  | Yogurt | IS inc. | 0.92 (0.86-0.98) |  |
|  |  |  |  | Cheese | IS inc. | 0.89 (0.81-0.97) |  |

| Author (Year) | country | Study period, Follow up (year) | Participants n, sex, Age (Years), number of cases/deaths | Exposure vs. comparator, subgroup | Outcome | RR or HR（95% CIs） | Adjustment |
| --- | --- | --- | --- | --- | --- | --- | --- |
|  |  |  |  | Eggs | IS inc. | 1.03 (0.95-1.12) |  |
|  |  |  |  | Cereals and cereal products | IS inc. | 0.94 (0.91-0.98) |  |
|  |  |  |  | Fruit | IS inc. | 0.94 (0.91-0.98) |  |
|  |  |  |  | Vegetables | IS inc. | 0.90 (0.82-0.99) |  |
|  |  |  |  | Legumes | IS inc. | 0.95 (0.88-1.03) |  |
|  |  |  |  | Nuts and seeds | IS inc. | 0.98 (0.87-1.11) |  |
|  |  |  |  | Red meat | HS inc. | 0.95 (0.77-1.16) |  |
|  |  |  |  | White fish | HS inc. | 1.04 (0.95-1.15) |  |
|  |  |  |  | Fatty fish | HS inc. | 0.96 (0.86-1.07) |  |
|  |  |  |  | Milk | HS inc. | 1.07 (0.99-1.16) |  |

| Author (Year) | country | Study period, Follow up (year) | Participants n, sex, Age (Years), number of cases/deaths | Exposure vs. comparator, subgroup | Outcome | RR or HR（95% CIs） | Adjustment |
| --- | --- | --- | --- | --- | --- | --- | --- |
|  |  |  |  | Yogurt | HS inc. | 0.94 (0.83-1.06) |  |
|  |  |  |  | Cheese | HS inc. | 0.88 (0.75-1.04) |  |
|  |  |  |  | Eggs | HS inc. | 1.25 (1.09-1.43) |  |
|  |  |  |  | Cereals and cereal products | HS inc. | 1.08 (0.78-1.49) |  |
|  |  |  |  | Fruit | HS inc. | 0.99 (0.93-1.06) |  |
|  |  |  |  | Vegetables | HS inc. | 1.04 (0.89-1.21) |  |
|  |  |  |  | Legumes | HS inc. | 1.06 (0.94-1.20) |  |
|  |  |  |  | Nuts and seeds | HS inc. | 0.90 (0.73-1.11) |  |

| Author (Year) | country | Study period, Follow up (year) | Participants n, sex, Age (Years), number of cases/deaths | Exposure vs. comparator, subgroup | Outcome | RR or HR（95% CIs） | Adjustment |
| --- | --- | --- | --- | --- | --- | --- | --- |
| B. Buijsse et al. (2010) | Germany | 1994-1998, ~8 years follow-up | 19357 M/F, age 35-65 years: 136 stroke cases | Chocolate consumption: Low vs. High | stroke inc. | 0.52 (0.30-0.89) | age, sex, alcohol intake, employment status, BMI, waist circumference, smoking, occupational physical activity, sports, cycling, education, total energy intake, prevalence of diabetes and for energy-adjusted intakes of fruit, vegetables, red meat, processed meat, dairy, coffee, tea, and cereal fibre |
| J.-Y. Dong et al. (2017) | Japan | cohort 1:1995 cohort 2: 1998, 12.9 years follow-up (median) | 84597 M/F, age 40-76 years old: 3558 stroke cases | Chocolate consumption: Low vs. High, M | stroke inc. | 0.94 (0.80-1.10) | age, area, BMI, smoking, sports, occupation, medication use for hypertension and hypercholesterolemia, and intakes of alcohol and total energy, intakes of green tea, coffee, fish/seafood, meat, fruits, soy foods and vegetable |
|  |  |  |  | Chocolate consumption: Low vs. High, F | stroke inc. | 0.84 (0.71-0.99) |  |
| Note: When multiple outcomes are present, we selected the risk ratios (RR) associated with the highest and lowest levels of intake. | | | | | | | |
| IS, ischemic stroke; HS, hemorrhagic stroke; BMI, body mass index; DASH, Dietary Approaches to Stop Hypertension; eGFR, estimated glomerular filtration rate; AMI, acute myocardial infarction | | | | | | | |

| **Table S3** Newcastle-Ottawa Scale for assessing the quality of studies in meta-analysis | | | |  |  |  |  |  |  |
| --- | --- | --- | --- | --- | --- | --- | --- | --- | --- |
|  | Selection | | | | Comparability | Outcome | | | Quality score |
| Study | Representativeness of the exposed cohort | Selection of the nonexposed cohort | Ascertainment of exposure | Demonstration that outcome of interest was not present at start of study | Comparability of cohorts on the basis of the design or analysis | Assessment of outcome | Was follow-up long enough for outcomes to occur^1^ | Adequacy of follow up of cohorts |  |
| Juan Juan et al. (2017) | 1 | 1 | 1 | 1 | 2 | 1 | 1 | 0 | 8 |
| I. Muraki et al. (2015) | 1 | 1 | 1 | 1 | 2 | 1 | 1 | 0 | 8 |
| E. S. Eshak et al. (2014) | 1 | 1 | 1 | 1 | 2 | 1 | 1 | 0 | 8 |
| A. M. Bernstein et al. (2012) | 1 | 1 | 1 | 1 | 2 | 1 | 1 | 1 | 9 |
| L. S. Pacheco et al. (2020) | 1 | 1 | 1 | 1 | 2 | 1 | 1 | 0 | 8 |
| S. Janzi et al. (2020) | 1 | 1 | 1 | 1 | 2 | 1 | 1 | 1 | 9 |
| N. Tanabe et al. (2008) | 1 | 1 | 1 | 1 | 2 | 1 | 1 | 0 | 8 |
| Y. Kokubo et al. (2013) | 1 | 1 | 1 | 1 | 2 | 1 | 1 | 0 | 8 |
| E. Lopez-Garcia et al. (2009) | 0 | 1 | 1 | 1 | 2 | 1 | 1 | 0 | 7 |
| D. E. Grobbee et al. (1990) | 0 | 1 | 1 | 1 | 2 | 1 | 0 | 1 | 7 |
| M. J. Stampfer et al. (1988) | 0 | 1 | 1 | 1 | 2 | 1 | 0 | 1 | 7 |
| S. Makita et al. (2012) | 0 | 1 | 1 | 1 | 2 | 1 | 1 | 0 | 7 |
| Q. Gao et al. (2021) | 1 | 1 | 1 | 1 | 2 | 1 | 1 | 0 | 8 |
| F. R. Scheffers et al. (2019) | 1 | 1 | 1 | 1 | 2 | 1 | 1 | 0 | 8 |
| L. S. Pacheco et al. (2022) | 1 | 1 | 1 | 1 | 2 | 1 | 1 | 0 | 8 |
| T. Yoshizaki et al. (2020) | 1 | 1 | 1 | 1 | 2 | 1 | 1 | 1 | 9 |

|  | Selection | | | | Comparability | Outcome | | | Quality score |
| --- | --- | --- | --- | --- | --- | --- | --- | --- | --- |
| Study | Representativeness of the exposed cohort | Selection of the nonexposed cohort | Ascertainment of exposure | Demonstration that outcome of interest was not present at start of study | Comparability of cohorts on the basis of the design or analysis | Assessment of outcome | Was follow-up long enough for outcomes to occur^1^ | Adequacy of follow up of cohorts |  |
| S. P. Johnsen et al. (2003) | 1 | 1 | 1 | 1 | 2 | 1 | 0 | 1 | 8 |
| V. Miller et al. (2017) | 1 | 1 | 1 | 1 | 2 | 1 | 1 | 1 | 9 |
| S. C. Larsson et al. (2016) | 1 | 1 | 1 | 1 | 2 | 1 | 1 | 0 | 8 |
| D. H. Lee et al. (2019) | 1 | 1 | 1 | 1 | 2 | 1 | 1 | 1 | 9 |
| S. Yu et al. (2023) | 1 | 1 | 1 | 1 | 2 | 1 | 0 | 0 | 7 |
| S. C. Larsson et al. (2018) | 1 | 1 | 1 | 1 | 2 | 1 | 1 | 0 | 8 |
| K. L. Ivey et al. (2021) | 1 | 1 | 1 | 1 | 2 | 1 | 0 | 0 | 7 |
| M. Guasch-Ferre et al. (2017) | 1 | 1 | 1 | 1 | 2 | 1 | 1 | 0 | 8 |
| H. Iso et al. (2001) | 0 | 1 | 1 | 1 | 2 | 1 | 1 | 0 | 7 |
| K. He, et al. (2002) | 0 | 1 | 1 | 1 | 2 | 1 | 1 | 0 | 7 |
| M. C. Morris et al. (1995) | 0 | 1 | 1 | 1 | 2 | 1 | 0 | 1 | 7 |
| M. Bonaccio et al. (2017) | 1 | 1 | 1 | 1 | 2 | 1 | 0 | 0 | 7 |
| S. Cui et al. (2022) | 1 | 1 | 1 | 1 | 2 | 1 | 0 | 0 | 7 |
| R. E. Ward et al. (2020) | 1 | 1 | 1 | 1 | 2 | 1 | 0 | 0 | 7 |
| A. Wallin et al. (2018) | 0 | 1 | 1 | 1 | 2 | 1 | 1 | 0 | 7 |
| P. Amiano et al. (2016) | 1 | 1 | 1 | 1 | 2 | 1 | 1 | 0 | 8 |

|  | Selection | | | | Comparability | Outcome | | | Quality score |
| --- | --- | --- | --- | --- | --- | --- | --- | --- | --- |
| Study | Representativeness of the exposed cohort | Selection of the nonexposed cohort | Ascertainment of exposure | Demonstration that outcome of interest was not present at start of study | Comparability of cohorts on the basis of the design or analysis | Assessment of outcome | Was follow-up long enough for outcomes to occur^1^ | Adequacy of follow up of cohorts |  |
| S. C. Larsson et al. (2011) F | 0 | 1 | 1 | 1 | 2 | 1 | 1 | 0 | 7 |
| N. Grau, N et al. (2022) | 1 | 1 | 1 | 1 | 2 | 1 | 1 | 0 | 8 |
| G. W. Dalmeijer et al. (2013) | 1 | 1 | 1 | 1 | 2 | 1 | 1 | 0 | 8 |
| J. Praagman et al. (2015) | 1 | 1 | 1 | 1 | 2 | 1 | 1 | 0 | 8 |
| K. Tanno et al. (2021) | 1 | 1 | 1 | 1 | 2 | 1 | 1 | 0 | 8 |
| M. Talaei et al. (2019) | 1 | 1 | 1 | 1 | 2 | 1 | 1 | 0 | 8 |
| J. R. Buendia et al. (2018) | 0 | 1 | 1 | 1 | 2 | 1 | 1 | 0 | 7 |
| S. C. Larsson et al. (2012) | 1 | 1 | 1 | 1 | 2 | 1 | 1 | 0 | 8 |
| T. Y. N. Tong et al. (2020) | 1 | 1 | 1 | 1 | 2 | 1 | 1 | 0 | 8 |
| F. B. Hu et al. (1999) | 1 | 1 | 1 | 1 | 2 | 1 | 1 | 0 | 8 |
| N. Mohammadifard et al. (2022) | 1 | 1 | 1 | 1 | 2 | 1 | 1 | 0 | 8 |
| S. C. Larsson et al. (2015) | 1 | 1 | 1 | 1 | 2 | 1 | 1 | 0 | 8 |
| L. Djousse et al. (2008) | 0 | 1 | 1 | 1 | 2 | 1 | 1 | 0 | 7 |
| A. M. Abdollahi et al. (2019) | 0 | 1 | 1 | 1 | 2 | 1 | 1 | 0 | 7 |
| B. Buijsse et al. (2010) | 1 | 1 | 1 | 1 | 2 | 1 | 0 | 1 | 8 |
| J.-Y. Dong et al. (2017) | 1 | 1 | 1 | 1 | 2 | 1 | 1 | 0 | 8 |
| ^1^: A follow-up period of greater than five years is considered to be adequate for the purposes of the study. | | | | | | | | | |

| **Table S4** GRADE evidence profiles for the association of dietary intakes and the risk of stroke | | | | | | | | | | | |
| --- | --- | --- | --- | --- | --- | --- | --- | --- | --- | --- | --- |
| The GRADE framework is a methodology for assessing the quality of evidence within systematic reviews, health technology assessments, and for grading recommendations throughout the healthcare sector. Using the GRADE system, the quality of evidence is categorized as very low, low, moderate, or high. In this evaluation, one reviewer (SY. H.) was responsible for initially assessing the certainty of the evidence, while a second reviewer (YC. F.) conducted a review and made necessary adjustments to the certainty assessments. The baseline level of evidence certainty was set to low, reflecting the inherent limitations associated with observational studies. Factors that led to a downgrade in evidence certainty included risk of bias—based on the Newcastle-Ottawa Scale (NOS) where studies with a score below 6 were considered to pose a risk—along with inconsistency, indirectness, imprecision, and publication bias. Inconsistency was determined by examining the similarity of point estimates, the overlap of confidence intervals, the direction of effects, with specific thresholds set at an I2 value of 50% or greater and a p-value less than 0.10. Indirectness was assessed by identifying factors that could restrict the applicability of the results.For determining imprecision, a threshold was set based on the literature, defining an optimal information size of 400 cases and 4000 participants, with a 25% relative risk (RR) reduction. Evidence was downgraded for imprecision if this threshold was not reached. Additionally, evidence was downgraded if the 95% confidence interval (CI) included 1.00, and if the upper and lower bounds of the 95% CI were less than 0.75 and greater than 1.25, respectively. Upgrades in evidence certainty were applied in cases where there was a large effect size (RR greater than 2 or less than 0.5 without plausible confounders), a dose-response gradient, or where there was evidence of opposing residual confounding. A GRADE evidence profile was subsequently developed to summarize these assessments. | | | | | | | | | | | |
| Certainty assessment | | | | | | | | Summary of findings | | | Certainty |
|  |  |  |  |  |  |  |  | No. of participants | | Effect |  |
| No. of studies | Study design | Risk of bias | Inconsistency | Indirectness | Imprecision | Publication bias | Other considerations | Participants | Cases | Relative (95% CI) |  |
| Cereal | | | | | | | | | | | |
| 2 | Observational studies | Not serious | Not serious | Serious^1^ | Not serious | NA^2^ | NA | 532902 | 9836 | 0.92 (0.87-0.98) | ⨁◯◯◯ VERY LOW |

|  | | | | | | | | | | | |
| --- | --- | --- | --- | --- | --- | --- | --- | --- | --- | --- | --- |
| Certainty assessment | | | | | | | | Summary of findings | | | Certainty |
|  |  |  |  |  |  |  |  | No. of participants | | Effect |  |
| No. of studies | Study design | Risk of bias | Inconsistency | Indirectness | Imprecision | Publication bias | Other considerations | Participants | Cases | Relative (95% CI) |  |
| Rice | | | | | | | | | | | |
| 2 | Observational studies | Not serious | Not serious | Serious^1^ | Not serious | NA^2^ | NA | 298779 | 9067 | 0.93 (0.84-1.03) | ⨁◯◯◯ VERY LOW |
| Fruits | | | | | | | | | | | |
| 8 | Observational studies | Not serious | Not serious | Serious^1^ | Not serious | NA^2^ | NA | 931853 | 24332 | 0.92 (0.89-0.96) | ⨁◯◯◯ VERY LOW |
| Vegetables | | | | | | | | | | | |
| 7 | Observational studies | Not serious | Not serious | Serious^1^ | Not serious | NA^2^ | NA | 879622 | 23615 | 0.97 (0.93-1.04) | ⨁◯◯◯ VERY LOW |
| Legume | | | | | | | | | | | |
| 3 | Observational studies | Not serious | Serious^3^ | Not serious | serious^3^ | NA^2^ | NA | 556141 | 6323 | 0.84 (0.65-1.09) | ⨁◯◯◯ VERY LOW |
| Coffee | | | | | | | | | | | |
| 3 | Observational studies | Not serious | Not serious | Not serious | Not serious | NA^2^ | NA | 210643 | 5759 | 0.81 (0.74-0.90) | ⨁⨁◯◯ LOW |
| Green tea | | | | | | | | | | | |
| 2 | Observational studies | Not serious | Serious | Serious^1^ | serious^3^ | NA^2^ | NA | 88336 | 3535 | 0.62 (0.30-1.28) | ⨁◯◯◯ VERY LOW |

| Certainty assessment | | | | | | | | Summary of findings | | | Certainty |
| --- | --- | --- | --- | --- | --- | --- | --- | --- | --- | --- | --- |
|  |  |  |  |  |  |  |  | No. of participants | | Effect |  |
| No. of studies | Study design | Risk of bias | Inconsistency | Indirectness | Imprecision | Publication bias | Other considerations | Participants | Cases | Relative (95% CI) |  |
| Sugar-sweetened beverages | | | | | | | | | | | |
| 3 | Observational studies | Not serious | Not serious | Serious^4^ | Not serious | NA^2^ | NA | 259511 | 12192 | 1.19 (1.09-1.30) | ⨁◯◯◯ VERY LOW |
| Alcohol | | | | | | | | | | | |
| 2 | Observational studies | Not serious | Not serious | Not serious | serious^3^ | NA^2^ | NA | 95540 | 306 | 0.93 (0.46-1.90) | ⨁◯◯◯ VERY LOW |
| Nuts | | | | | | | | | | | |
| 4 | Observational studies | Not serious | Serious | Serious^4^ | Not serious | NA^2^ | NA | 870356 | 20711 | 0.93 (0.84-1.03) | ⨁◯◯◯ VERY LOW |
| Fish | | | | | | | | | | | |
| 8 | Observational studies | Not serious | Not serious | Serious^1^ | Not serious | NA^2^ | NA | 841680 | 13969 | 0.99 (0.94-1.04) | ⨁◯◯◯ VERY LOW |
| Red meat | | | | | | | | | | | |
| 5 | Observational studies | Not serious | Serious | Serious^1^ | Not serious | NA^2^ | NA | 539742 | 12298 | 1.00 (0.86-1.16) | ⨁◯◯◯ VERY LOW |
| Eggs | | | | | | | | | | | |
| 5 | Observational studies | Not serious | Not serious | Serious^1^ | Not serious | NA^2^ | NA | 216148 | 6804 | 1.07 (1.00-1.14) | ⨁◯◯◯ VERY LOW |

|  | | | | | | | | | | | | |
| --- | --- | --- | --- | --- | --- | --- | --- | --- | --- | --- | --- | --- |
| Certainty assessment | | | | | | | | | Summary of findings | | | Certainty |
|  |  |  |  |  |  |  |  |  | No. of participants | | Effect |  |
| No. of studies | Study design | Risk of bias | Inconsistency | Indirectness | Imprecision | Publication bias | | Other considerations | Participants | Cases | Relative (95% CI) |  |
| Cheese | Cheese | Cheese | Cheese | Cheese | Cheese | Cheese | | Cheese | Cheese | Cheese | Cheese | Cheese |
| 4 | Observational studies | Not serious | Not serious | Serious^1^ | Not serious | NA^2^ | | NA | 531150 | 12562 | 0.92 (0.87-0.97) | ⨁◯◯◯ VERY LOW |
| Other fermented dairy | | | | | | | | | | | | |
| 5 | Observational studies | Not serious | Not serious | Serious^1^ | Not serious | NA^2^ | | NA | 605032 | 14828 | 0.94 (0.90-0.98) | ⨁◯◯◯ VERY LOW |
| Milk | | | | | | | | | | | | |
| 5 | Observational studies | Not serious | Not serious | Serious^1^ | Not serious | NA^2^ | | NA | 517078 | 12650 | 0.95 (0.92-0.99) | ⨁◯◯◯ VERY LOW |
| Chocolate | | | | | | | | | | | | |
| 2 | Observational studies | Not serious | Serious | Not serious | serious^3^ | NA^2^ | | NA | 103954 | 3694 | 0.73 (0.44-1.21) | ⨁◯◯◯ VERY LOW |
| CI, Confidence interval | | | | | | | | | | | | |
| ^1^: Downgraded by one level because all studies were conducted in Western countries. | | | | | | | | | | | | |
| ^2^: Publication bias could not be assessed due to limited number of studies. | | | | | | | | | | | | |
| ^3^: I^2^＞50% and p<0.05 in heterogeneity test. | | | | | | | | | | | | |
| ^4^: Downgraded due to the indirect nature of comparing intake frequency with quantity consumed. | | | | | | |  |  |  |  |  |  |


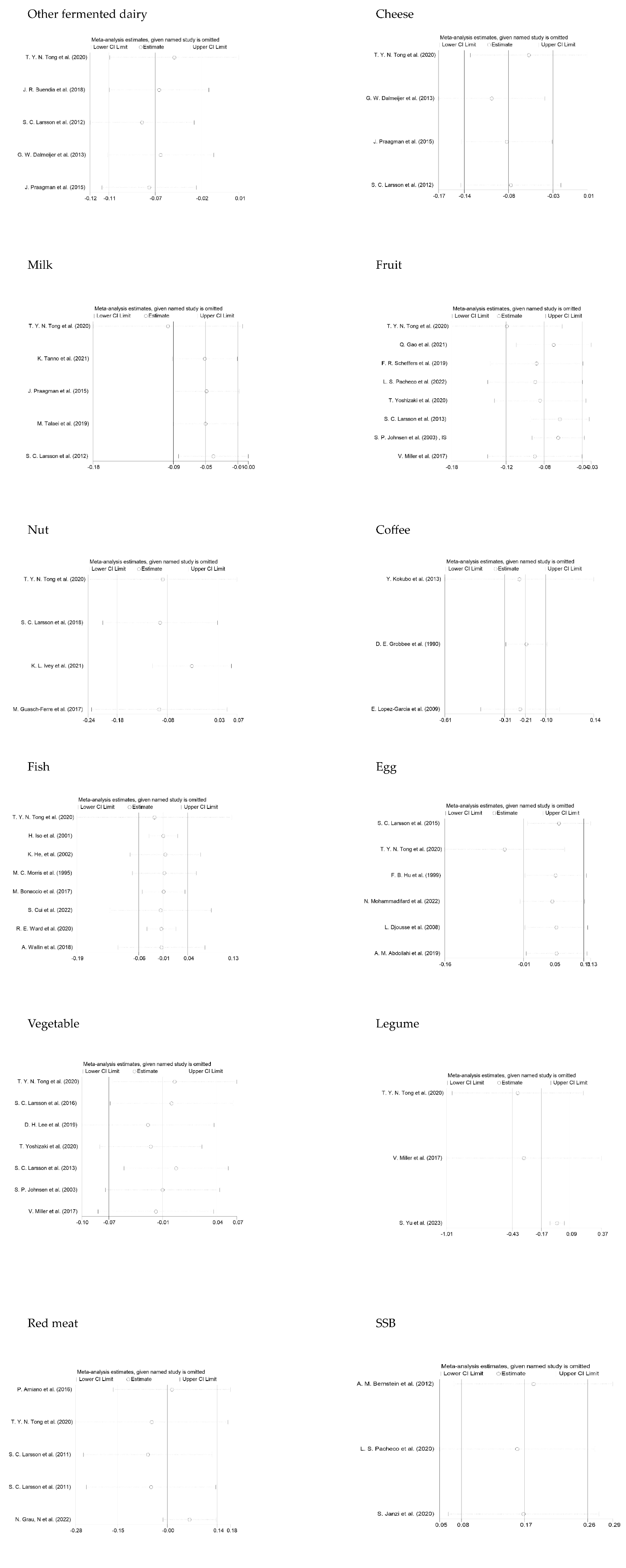


**Figure S1** Sensitivity Analysis of Meta-Analysis Results for Dietary Intake and Stroke.


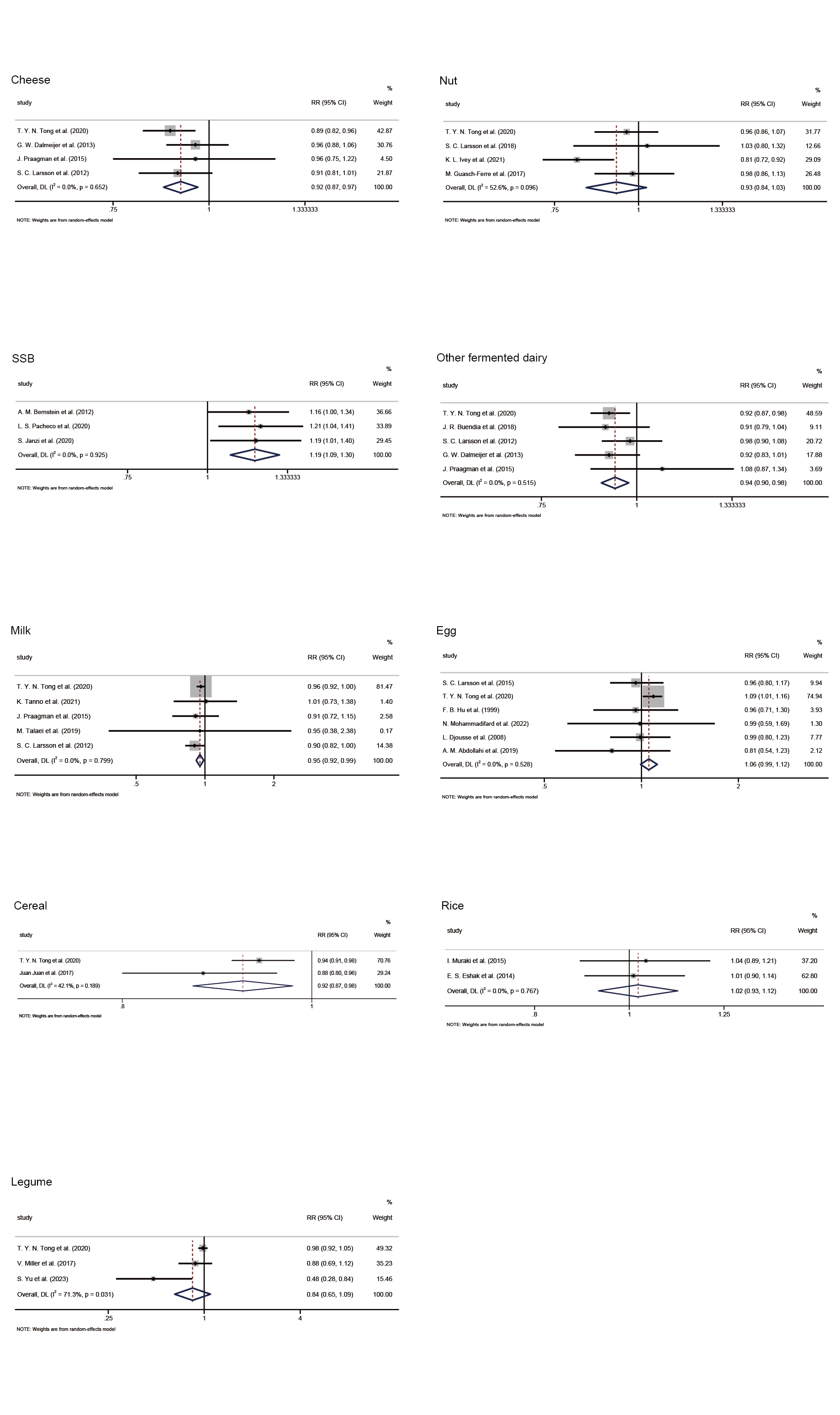


**Figure S2** Meta-analysis forest plot.
